# Glacial legacies on interglacial vegetation at the Pliocene-Pleistocene transition in NE Asia

**DOI:** 10.1038/ncomms11967

**Published:** 2016-06-24

**Authors:** Ulrike Herzschuh, H. John B. Birks, Thomas Laepple, Andrei Andreev, Martin Melles, Julie Brigham-Grette

**Affiliations:** 1 Periglacial Research Section, Alfred Wegener Institute, Helmholtz Centre for Polar and Marine Research, Telegraphenberg A43, 14473 Potsdam, Germany; 2 Institute of Earth and Environmental Sciences, Faculty of Sciences, University Potsdam, 14479 Potsdam-Golm, Germany; 3 Department of Biology, University of Bergen, Postboks 7803, 5020 Bergen, Norway; 4 Bjerknes Centre for Climate Research, Bergen, Norway; 5 Environmental Change Research Centre, University College London, London WC1E 6BT, UK; 6 Institute of Geology and Mineralogy, Faculty of Mathematics and Natural Sciences, University of Cologne, 50674 Köln (Cologne), Germany; 7 Institute of Geology and Petroleum Technologies, Faculty of Natural Sciences, Kazan Federal University, Kazan 420008, Russia; 8 Department of Geosciences, University of Massachusetts Amherst, Amherst, Massachusetts 01003-9297, USA

## Abstract

Broad-scale climate control of vegetation is widely assumed. Vegetation-climate lags are generally thought to have lasted no more than a few centuries. Here our palaeoecological study challenges this concept over glacial–interglacial timescales. Through multivariate analyses of pollen assemblages from Lake El'gygytgyn, Russian Far East and other data we show that interglacial vegetation during the Plio-Pleistocene transition mainly reflects conditions of the preceding glacial instead of contemporary interglacial climate. Vegetation–climate disequilibrium may persist for several millennia, related to the combined effects of permafrost persistence, distant glacial refugia and fire. In contrast, no effects from the preceding interglacial on glacial vegetation are detected. We propose that disequilibrium was stronger during the Plio-Pleistocene transition than during the Mid-Pliocene Warm Period when, in addition to climate, herbivory was important. By analogy to the past, we suggest today's widespread larch ecosystem on permafrost is not in climate equilibrium. Vegetation-based reconstructions of interglacial climates used to assess atmospheric CO_2_–temperature relationships may thus yield misleading simulations of past global climate sensitivity.

## Abstract (web summary)

Climate is broadly assumed to control vegetation, with vegetation lags thought to last no more than a few centuries. Here, based on the analysis of Lake El'gygytgyn pollen record, the authors show that vegetation-climate disequilibrium persisted for several millennia during the Plio-Pleistocene transition.

## Graphical abstract

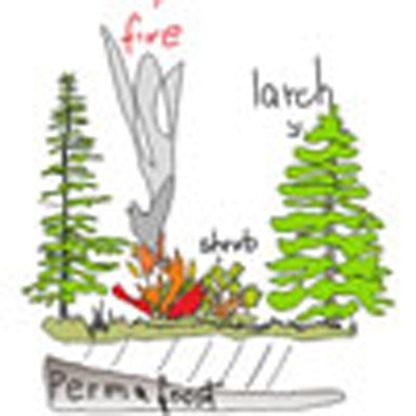

Natural vegetation patterns in space and time are assumed to reflect mainly climate variability on all environmentally relevant temporal and spatial scales^1^,^2^. For example, it has been shown that vegetation turnover in space can substitute for turnover in time on millennial timescales^3^, which fits with the findings that the climatic niches of individual plant taxa are generally stable over even longer timescales^4^. Although lag times between vegetation and climate changes have been extensively discussed with respect to tree migration patterns in Europe and North America following the last glacial stage^5^,^6^,^7^, most proxy studies do not suggest major vegetation–climate disequilibria^8^. Relevant model evidence is lacking. Patterns of Late Pleistocene interglacial vegetation histories (marine isotope stage (MIS) 11, 9, 7 and 5) as inferred from long-term pollen records from Greece^9^ and the Massif Central^10^ are, in addition to their established relationship with interglacial climate, hypothesized to reflect the different locations of glacial refugia, creating unique interglacial migrational patterns. This accords with the suggestion that in addition to climate, post-glacial migration limitations might also explain the current distribution of many European plant species^11^. Furthermore, an analysis integrating modern plant community composition, their climatic niches and climate change since the Last Glacial Maximum implies that North and South American forests are still responding to last glacial climate change^12^. However, systematic investigations of changes in vegetation–climate relationships over glacial-interglacial timescales are lacking and thus the concept of ‘glacial legacies' on interglacial vegetation has not been demonstrated using proxy data.

High-northern latitudes are ideally suited to detect former non-equilibrium patterns as climate change is particularly strong due to polar amplification^13^. Furthermore, the southern expansion of permafrost soils during glacial climate states^14^ may have increased the distance between glacial refugia and potential interglacial refugial areas. However, non-climatic vegetation drivers such as herbivory^15^,^16^, fire^17^ or soil disturbance^18^ should also be considered. Finally, tectonic impacts and their climatic effects may represent a hidden driver of long-term vegetation change in the Arctic^19^.

The only high-latitude pollen record continuously spanning several glacial-interglacial cycles is from Lake El'gygytgyn in Chukotka, Russian Far East ranging from 3,530 to 2,150 kyr ago BP (refs 20, 21), covering both the Mid-Pliocene Warm Period (MPWP: 3,530–2,900 kyr ago) and the Plio-Pleistocene transition (PPT: 2,900–2,150 kyr ago). The identification of vegetation–climate disequilibrium requires proxy records that reliably reflect regional climate change and are independent of the Lake El'gygytgyn pollen data and of sufficient temporal length and resolution. Unfortunately, no independent climate record from Lake El'gygytgyn or any other arctic terrestrial site of sufficient data quality exists. Accordingly, only marine records can be used. Here we assume and later confirm that the globally integrating benthic isotope stack (LR04)^22^ and nearest sea-surface temperature (SST)^23^ records from the Pacific can be used to approximate the climate conditions at our study site. While all climate records indicate that the relative intensities of glacials and interglacials vary over time, a behaviour likely related to differences in orbital configuration^24^ and internal climate dynamics, these intensities appear to be largely globally coherent.

To assess the impact of herbivory, fire and soil disturbance on vegetation, common non-pollen palynomorphs in lake sediments can be used including *Sporormiella*^25^ (a coprophilous fungal spore), *Gelasinospora*^26^ (a fungal spore characteristic of burned soils) and *Glomus*^27^,^28^ (an endomycorrhizal fungal spore characteristic of disturbed soils), respectively.

Here through multivariate analyses we show that variation among interglacial pollen assemblages at Lake El'gygytgyn through the PPT is best explained by long-term drivers (that is, proxies for climate and the vegetation condition of the preceding glacial), which we interpret to indicate major vegetation–climate disequilibrium. In contrast, short-term drivers (that is, proxies for contemporaneous climate, soil disturbance/erosion and/or herbivory) best explain statistically the variations in the pollen data of the PPT glacial and MPWP interglacials and glacials. From these results we conclude that the severe glacial climate and related extensive permafrost during some PPT led to distant glacial refugia, particularly of evergreen tree taxa, that hindered their subsequent interglacial establishment and the development of forests attaining equilibrium with climate. Our results imply that the current widespread larch ecosystem actually represents a transitional vegetation type reflecting severe last glacial (MIS2) conditions rather than contemporaneous Holocene interglacial (MIS1) climate.

## Results

### Vegetation change on glacial–interglacial timescales

**Figure 1:**
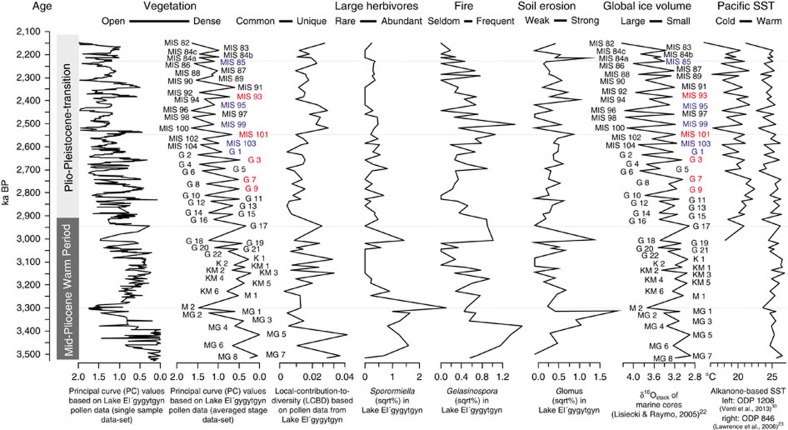
Proxy records of vegetation change and of changes of potential vegetation drivers during the MPWP and the PPT. Stratigraphic plot including the principal curve of the original pollen data set and of the averaged stage data set, and the Local-Contribution-to-Beta-Diversity (LCBD) of each stage, all based on analyses of the Lake El'gygytgyn pollen data. It also includes environmental data of inverted globally averaged oxygen stable isotope measurements of benthic foraminifera (δ^18^O_stack_)^22^, SST of ODP1208 (mid-latitude Pacific)^30^ and ODP846 (Tropical Eastern Pacific)^23^, and *Sporormiella* sqrt% (herbivory indicator), *Gelasinospora* sqrt% (fire indicator) and *Glomus* sqrt% (soil disturbance/erosion indicator) relative to the vascular-plant pollen sum from Lake El'gygytgyn^21^. The names of the glacials and interglacials are indicated. Red and blue labels indicate PPT interglacials with preceding weak and strong glacials, respectively, that were included in the comparison of inter-taxa relationships (see text and Fig. 5). Dotted horizontal lines are drawn to aid visual comparisons. sqrt, square root.

**Figure 2:**
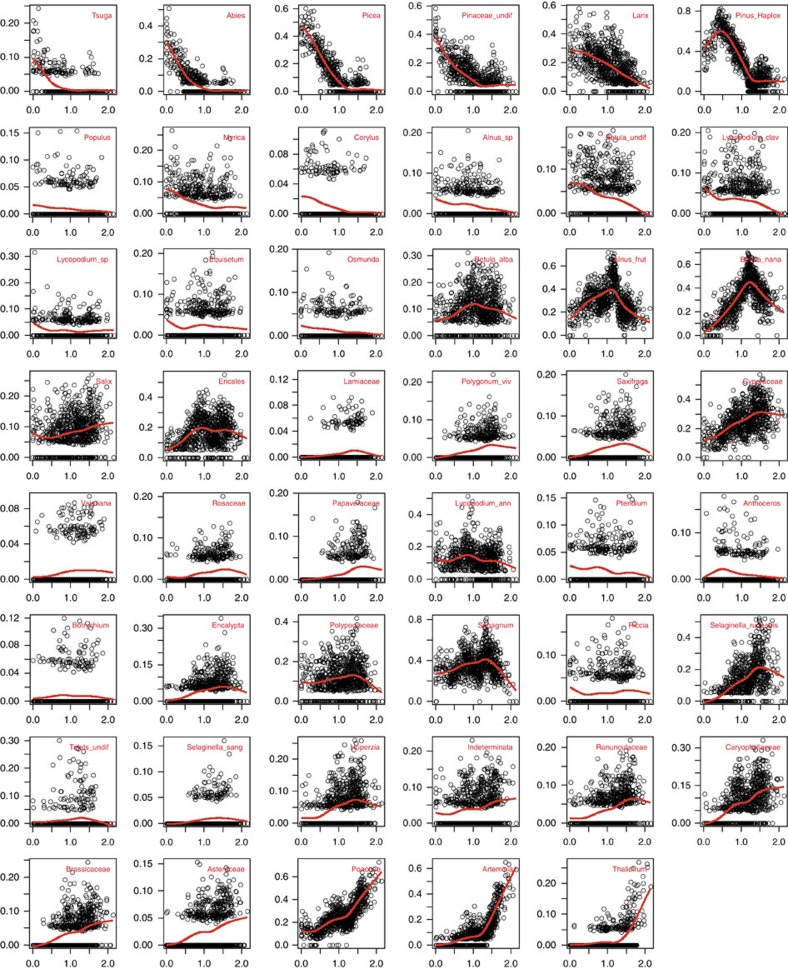
Fitted response curves of each pollen or spore taxon along the principal curve. Open circles are the observed Hellinger-transformed abundance along their principal curve locations, and the solid red line is the optimized smoother from the final iteration of the principal curve. The taxa are arranged according to visual inspection of their response curves' optima (from right to left and from top to bottom). The vertical axis is the Hellinger-transformed pollen taxon abundance (%) and the horizontal axis is distance along the principal curve.

A principal curve of the Lake El'gygytgyn pollen record, which summarizes complex multivariate data in one dimension, shows strong temporal variations (Fig. 1 and Supplementary Figs 1 and 2, 47% variation explained). The most obvious turnover at the glacial–interglacial timescale has already been described and related to 41 kyr obliquity insolation cyclicity^20^. Furthermore, the principal curve is consistent with a landscape openness curve based on the pollen-based biome-reconstruction technique of the same pollen record^29^. This suggests that millennial-scale vegetation variability generally reflects global climate shifts between glacial and interglacial states known from many proxy-based reconstructions^22^,^23^,^30^. The sequence of pollen types along the principal curve (Fig. 2) generally reflects their present-day relative occurrences in vegetation ranging from arctic steppe-tundra to dark needle-leaf forests^31^. As we focus on variability at glacial–interglacial timescales, similar principal curve analyses were performed with pollen spectra averaged for each marine isotope stage (see Methods). They show a very similar trend (Fig. 1, 75% variation explained: Supplementary Fig. 3). Inferred glacial–interglacial vegetation variability generally confirms SST reconstructions^23^, suggesting that the glacial–interglacial amplitude during the MPWP was rather low compared with that of the PPT. Furthermore, the principal curve suggests that differences in pollen composition among glacials and among interglacials are in the same range as glacial–interglacial variability, particularly during the PPT. As expected, the pollen spectra-specific contribution to compositional turnover (=Local-Contribution-to-Beta-Diversity^32^), here used as a measure of the uniqueness of the pollen composition in a marine isotope stage, is generally high for stages with extreme principal curve values.

### A globally coherent pattern of stage intensities

**Figure 3:**
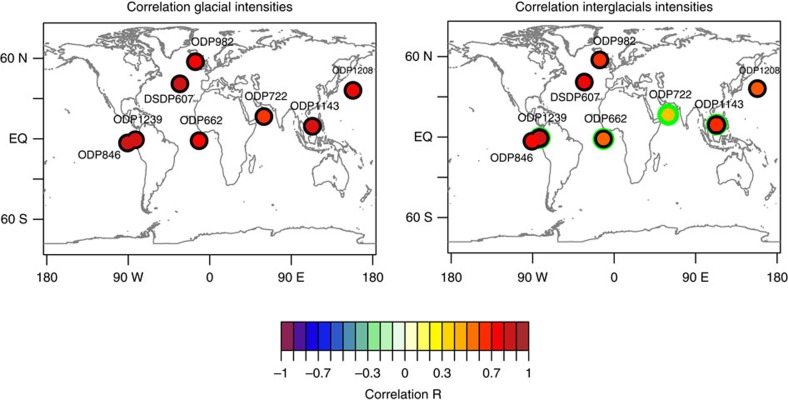
Correlation of glacial and interglacial intensities of marine UK^33^ temperature records with the inverted LR04 stack. All correlations are calculated during the core period (2,150–2,900 kyr ago), except ODP-982, which is evaluated for 2,500–3,500 kyr ago as the data resolution between 2150 and 2500 kyr ago does not allow determination of the minima and maxima. Black circles indicate significance at *P*=0.05; green circles indicate records with interglacial peaks near the limit of UK^33^ thermometry (>27 °C). (See Supplementary Fig.4 and Supplementary Table 1 for details and references of records.)

To test our assumption that we can approximate the climate conditions at our site with remote marine temperature and isotope records, we first investigate the global pattern of glacial and interglacial intensities. We compare the time series of glacial peak temperatures of a global set of SST records (Supplementary Table 1 and Supplementary Fig. 4) with the glacial intensities recorded in the LR04 stack (see Methods). The same analysis is also performed on the interglacial intensities. To ensure that most of the records overlap, we analyse the correlation during the PPT (2,150–2,900 kyr ago). The glacial intensities in all records cores show a significant (*P*<0.05) positive correlation to the inverted LR04 stack (Fig. 3) with a mean correlation of *r*=0.81. Interglacial intensities also show a positive correlation at all core sites with the inverted LR04 stack (mean *r*=0.69). The weakest correlations are obtained for the tropical cores from the Indian Ocean (ODP722) and Atlantic (ODP662). As the interglacial temperatures at these cores are also at the temperature limit of the specific (UK^33^) proxy (Supplementary Table 1), it is unclear if this is a climatic signal or a proxy artefact.

While a systematic Plio-Pleistocene analysis, omitting pollen-based records to avoid circular reasoning, is limited to marine temperature proxies, analysing the Late Quaternary period also allows the inclusion of other climate proxy records. Here Antarctic ice cores provide independent evidence for the coherency of glacial–interglacial intensities^34^. Our comparison of stage extremes in isotope, CO_2_ and methane records (Supplementary Fig. 5) show a significant correlation of the glacial intensities (mean correlation of ice-core records with the LR04 stack *r*=0.52) and a very strong correlation of the interglacial intensities (*r*=0.91). This result strongly suggests that the synchronizing factors (including greenhouse gases) are dominant over the local imprint of seasonal insolation^24^ in shaping the strength of the interglacials.

### Drivers of vegetation change

**Figure 4:**
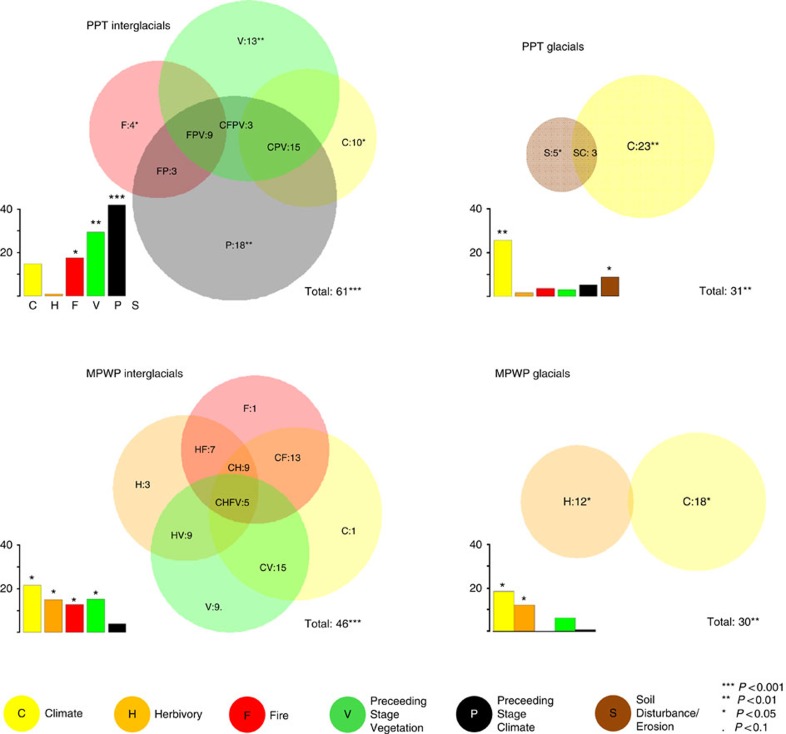
Drivers of vegetation change of interglacials and glacials of the MPWP and the PPT. Bar charts indicating the variation of the pollen spectra explained by single environmental variables (climate (C), herbivory (H), fire (F), preceding-stage climate (P), preceding-stage vegetation (V) and soil disturbance erosion (S)) for each of the analysed data sets for glacials and interglacials of the PPT and of the MPWP. Venn diagrams indicate the results of variation partitioning with those variables included that separately explain a statistically significant part of the variation (*P*<0.1). Total variation explained (in %) is indicated in the lower left part of each sub-plot (see Supplementary Table 2 and Supplementary Fig. 6/7 for further detailed RDA results). Results imply that vegetation conditions of interglacials of the PPT are best explained by long-term drivers such as the climate and vegetation condition of the preceding glacial, instead of contemporaneous conditions such as climate or herbivory. In contrast, vegetation conditions of the PPT glacials and MPWP interglacials can be best explained by short-term drivers. Accordingly, we assume that vegetation conditions of interglacials with preceding cold glacials were in strong vegetation–climate disequilibria.

To separate the effects of long-term and short-term drivers on interglacial vegetation, redundancy analysis (RDA) was applied to the data set of PPT averaged interglacial pollen spectra and proxy-based environmental variables (Fig. 1). We find that variables reflecting contemporaneous interglacial climate and fire, that is, short-term ecological drivers, explain only a small portion of the variation in the pollen data when included separately in RDA (15% *P*<0.1, 18% *P*<0.05, respectively, Fig. 4 and Supplementary Table 2) compared with variables reflecting climate and vegetation type from the preceding glacial stage (42% *P*<0.001, 29% *P*<0.01, respectively). Though a large part of pollen variation is shared by all or is a combination of several variables, each variable explains a unique portion of variation as inferred from variation partitioning using a specific ensemble of RDA runs (Fig. 4 and Supplementary Figs 6 and 7). Preceding glacial climate explains the highest unique portion (18%) of pollen variation.

To test whether this result is sensitive on the choice of the climate record, RDAs of PPT interglacial pollen assemblages were repeated for each of the globally available climate records (Supplementary Table 1) with reasonable data quality. Results (Supplementary Table 3) reveal that the preceding glacial climate in all tested cases explains a significant unique portion while interglacial climate does not explain a unique portion or only a small portion. Consistent with the global coherency of the glacial–interglacial intensities, this indicates that neither the selection of a specific proxy type nor the selection of a specific site would impact the major outcome of this study.

In addition, similar RDAs were applied to the other averaged data sets. We find that the variation in the PPT glacial pollen spectra is most parsimoniously explained by the contemporary climate variable and to a lesser extent by the soil disturbance/erosion proxy, with both explaining a unique and statistically significant portion of the variation (Fig. 4). MPWP interglacial pollen are significantly correlated to climate, vegetation of the preceding glacial, herbivory and fire when each variable (or variable set) is considered separately in RDA. However, variation partitioning shows that the major part of the explained variation is shared by all or a combination of several variables. MPWP glacial pollen spectra are most strongly related to contemporaneous glacial climate and herbivory (Fig. 4).

### Differences among PPT interglacial vegetation histories

**Figure 5:**
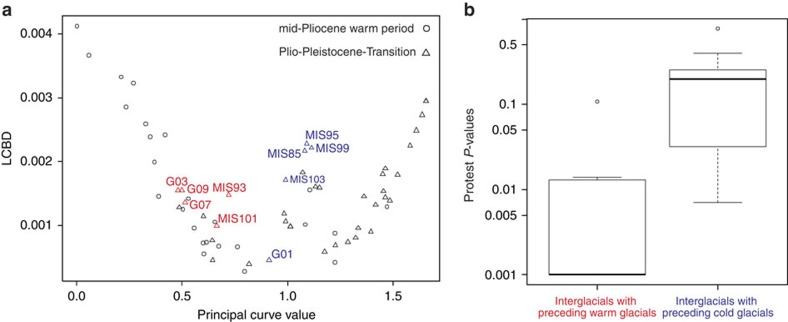
Comparison of pollen-based vegetation characteristics of PPT interglacials with preceding weak glacial conditions to PPT with preceding strong glacial conditions. (**a**) Principal curve values versus Local-Contribution-to-Beta-Diversity (LCBD) values for all MPWP and PPT interglacials and glacials. Red labels denote interglacials with a preceding warm glacial (G09, G07, G03, MIS101 and MIS93), and blue labels denote interglacials with preceding cold glacials (G01, MIS103, MIS99, MIS95 and MIS85). It is clear that averaged pollen spectra of interglacials with preceding cold glacials strongly contribute to the LCBD when compared with spectra from other stages with similar principal curve values, in contrast to averaged spectra from interglacials with preceding warm glacials. (**b**) Boxplots of *P* values obtained for pairwise Procrustes comparison of ordination-derived variable scores of 13 taxa among 5 selected PPT interglacials with preceding weak (warm) glacials and among 5 selected PPT interglacials with preceding strong (cold) glacials. Whiskers represent the 1.5 time length of interquartile range. Results indicate that inter-taxa relationships among interglacials with a preceding cold glacial differ strongly among each other (that is, indicated by high Protest *P* values) while they show a significant fit among interglacials with preceding warm glacials. We assume that the vegetation histories of interglacials with preceding cold glacials were unique as a result of distant glacial refugia causing broad-scale vegetation-climate disequilibria.

On the basis of the RDA biplots (Supplementary Fig. 6a,b) where variables for the preceding glacial are constrained to the first RDA axes and all the remaining known environmental variation is partialled out, we conclude that interglacials with preceding weak glacials and prevailing tree-tundra, such as G9, G7, G3, MIS101 and MIS93, are characterized by *Pinus* (pine) and *Picea* (spruce) forests (see also interglacial sequences in Supplementary Figs 8 and 9). Furthermore, these interglacials are closely located in the principal curve versus Local-Contribution-to-Beta-Diversity plot (Fig. 5a). Interglacials with preceding warm glacials are also characterized by similar inter-taxa relationships as inferred from the statistically significant fit of all interglacial pairs of ordination-derived taxa scores shown by Procrustes analyses and associated Protests (see Methods, Fig. 5b, see also taxa-specific residuals in Supplementary Fig. 10). This suggests that they reflect similar interglacial vegetation histories. In summary, during the PPT, interglacials with preceding warm glacial stages share similar evergreen forest vegetation, which we assume is driven by and is in equilibrium with the contemporaneous climate.

In contrast, *Pinus* and *Picea* are absent or rarely occur during interglacials with a preceding cold glacial-stage climate and extensive arctic steppe such as G1, MIS103, MIS99, MIS95 and MIS87 while deciduous shrubs (in particular *Alnus* (alder)) and wetlands (indicated by high Cyperaceae (sedge) and *Sphagnum* (bog-moss) values) are abundant (Supplementary Figs 2, 8 and 9; see also taxa-specific residuals in Supplementary Fig. 10). Some of these interglacial spectra strongly contribute to the Local-Contribution-to-Beta-Diversity when compared with spectra from other stages with similar principal curve values (Fig. 5a). Also, the inter-taxa relationships differ strongly among single interglacials as indicated by high Protest *P* values (Fig. 5b and Supplementary Figs 8 and 9). In summary, interglacials with preceding strong (cold) glacial stages lack *Pinus* and *Picea* and their vegetation characteristics are non-analogous among each other.

## Discussion

With respect to PPT interglacials, we infer from our analyses of the Lake El'gygytgyn pollen record that the environmental state of the preceding glacial was more relevant for subsequent interglacial vegetation development, in particular for the presence or absence of evergreen conifers, than the contemporaneous interglacial climate, contradicting the hypothesis of a close vegetation–climate equilibrium. One alternative explanation for the observed weak relationship between proxy series for interglacial climate and interglacial pollen data would be that the analysed δ^18^O_stack_ and mid-latitude Pacific SST do not represent the regional interglacial climate around Lake El'gygytgyn due to proxy-specific or regional-specific differences. However, our analysis shows that the strength of an interglacial is largely coherent in the PPT between all available high-quality SST records from the Northern Hemisphere (Fig. 3), and is coherent between different proxy records, including Antarctic temperature and greenhouse gases in the last 800 kyr (Supplementary Fig. 4). This is consistent with theoretical and model considerations that climate variations on long timescales are typically associated with broad spatial scales^35^,^36^ and with evidence from climate-model simulations using orbital forcing as well as greenhouse-gas forcing^37^.

Such a ‘local climate' hypothesis would also be inconsistent with our finding that the climate of the preceding glacial as recorded by the δ^18^O_stack_ and mid-latitude Pacific SST should influence the local vegetation of the interglacial. Hence, we conclude that the most parsimonious explanation for our results is that glacial climate and vegetation conditions are the main drivers of interglacial vegetation.

**Figure 6:**
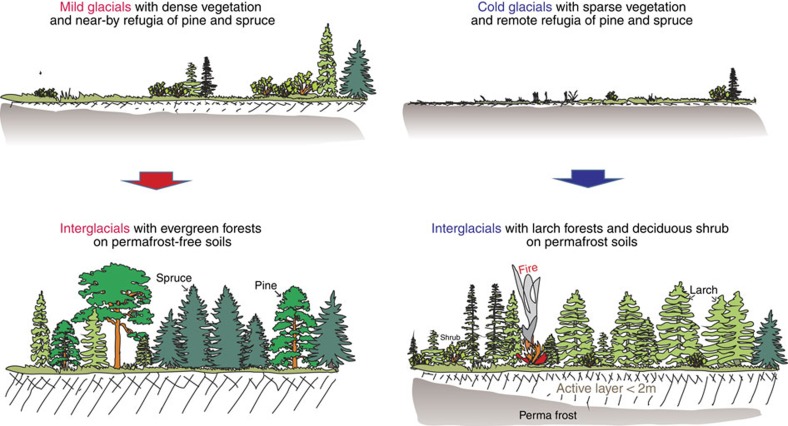
Visualization of the conceptual model of the ‘Glacial legacy on interglacial vegetation'. We assume that interglacial vegetation during the PPT mainly reflects conditions of the preceding glacial instead of contemporary interglacial climate. During interglacials following mild glacials, NE Asia was colonized by evergreen trees from nearby refugia. In contrast, during interglacials following cold glacials larch and deciduous shrubs expand in the area related to the combined effects of permafrost persistence and distant glacial refugia of evergreen trees, and fire. This implies that vegetation–climate disequilibrium can last for many millennia.

Accordingly, we interpret the absence of *Pinus* and *Picea* in Lake El'gygytgyn pollen spectra from interglacials following strong glacial stages to be related to three main factors: permafrost extent, distant glacial refugia, and extensive fires, plus possible interactions (Fig. 6). Today the vast continuous permafrost zone in NE Asia is covered by mono-specific larch forests with a deciduous-shrub understorey^9^. Occurrences of evergreen taxa (for example, *Picea obovata* and *Pinus sibirica*: for *Pinus pumila* see Supplementary Note 1) are restricted to the southern and western permafrost limit characterized by a summer thaw depth >1.5 m and azonal vegetation, such as on deep soils in river beds^9^. Permafrost with a shallow active layer is also assumed to limit the northward extent of *Picea* in Alaska^33^. In contrast, *Larix gmelinii* and *Larix cajanderi* can grow on soils with an active-layer depth of <40 cm (ref. 38). Thus, the question arises whether glacial-stage permafrost conditions are able to persist through strong interglacial warming and hence hinder or slow down the invasion of evergreen taxa and are therefore the key environmental link causing vegetation–climate disequilibrium. It can be assumed that permafrost during strong glacials was particularly extensive and deep because widespread continental-scale ice sheets were absent here and would not insulate soils from the very cold and harsh glacial climate^39^. Furthermore, during strong glacials, permafrost was likely more widespread due to enhanced continentality related to the absence of in-land moisture transport (that is, no snow in winter), perhaps as a result of lower sea level (60–70 m)^40^ and more persistent sea-ice over the Arctic ocean^41^. Though permafrost was inferred to persist even to a few metres below the surface in warmer climates during the Eemian interglacial in areas that are today located in the discontinuous permafrost zone^42^, modelling results indicate that the active-layer depth responds rather directly to climate^43^ while only deep permafrost may have millennial-scale lags to insolation maxima^44^. However, model studies have not been performed over long timescales and most do not include all relevant Earth-system components and their interactions. Our knowledge of permafrost response to climate change during the PPT is thus very limited.

The more southerly extensive permafrost during strong glacial stages possibly shifted the glacial refugia of *Pinus* and *Picea* farther south in addition to the severe glacial climate. For example, the vast steppes recorded at Lake El'gygytgyn during MIS96 stretched as far south as the Lake Baikal region, which accompanied the widespread extinction of evergreen needle-leaf taxa^45^. In contrast, *Larix* (larch) probably survived in NE Russia during all glacials, by analogy to its last glacial maximum (LGM) history^46^. The pollen compositional turnover of the PPT interglacials indicates the establishment of dense larch forests with shrub *Alnus* (Supplementary Fig. 9). These forests in particular, when occurring in combination with a thick *Sphagnum* cover^47^, insulate the soil from climate, thereby stabilizing the permafrost^38^ and likely hinder the germination and establishment of *Picea* and *Pinus*. Such an ecosystem with a high-standing biomass in a warm, continental early interglacial climate is likely to experience frequent fires. This is indicated by the maximum *Gelasinospora* abundance coincident with the *Larix*–*Alnus*-type optimum in the interglacial sequences (Supplementary Fig. 9). By a study of succession in present forests from central Siberia it has been shown that the ratio of deciduous (*Larix* and short-lived shrubs) to evergreen taxa cover (*Picea* and *Pinus*) is higher at sites characterized by frequent fires^48^. Hence it is reasonable to assume that no single driver caused the expansion of deciduous shrubs and larch forests in warm interglacials but the combination of persistent permafrost, distant refugia and high fire frequency may have slowed the invasion of *Picea* and *Pinus* and thus caused long-term vegetation–climate disequilibrium (Fig. 6).

The prevailing transient vegetation types during interglacials following strong glacials have unique characteristics, which probably originated from taxon reshuffling. In contrast, during interglacials following weak glacial stages, *Pinus* and *Picea* remained in the area or in nearby refugia and invaded northern Siberia and the northern Russian Far East before shrub-rich larch forests became stabilized by feedbacks with permafrost and fire. Accordingly, vegetation–climate equilibrium, characterized by a fixed vegetation composition, was reached in a consistent manner. Hence, we conclude that disequilibrium versus equilibrium conditions differentiate interglacials with strong or weak preceding glacial states rather than a bifurcation with two stable states.

Our reasoning that permafrost presence likely contributed to the slow invasion of evergreen taxa is further supported by results from numerical analyses with the remaining averaged data sets. We find that the effect of preceding glacial drivers is much smaller during MPWP interglacials when widespread permafrost was probably absent. In contrast, contemporaneous climate during the MPWP was a main driver. For example, the warm climate supported the expansion of dark needle-leaf forests during MPWP interglacials, in particular during the two oldest recorded interglacials (MG7 and MG5) that were also among the warmest with the smallest ice-sheet extent^22^,^23^. Furthermore, MPWP and PPT glacial vegetation is not related to interglacial climate or vegetation conditions but is best explained statistically by contemporaneous climate. In other words, severe climate change during a glacial-to-interglacial transition causes a leading-edge disequilibrium due to slow invasion^8^, while a slow die-back of long-lived trees (trailing-edge disequilibrium) is unlikely given the severe climate deterioration during an interglacial–glacial transition; the Arctic is known for its strong feedback mechanisms^13^.

In addition to long-term drivers, our study provides evidence that short-term drivers can disturb the vegetation–climate relationship on glacial–interglacial timescales. Our analyses suggest that MPWP glacial and interglacial vegetation is significantly related to changes in herbivory (Fig. 4). It is assumed that large herbivores, particularly mammoths and mastodons but also deer and bison, have the potential to act as ‘ecological keystones' in high-latitude vegetation^15^,^16^,^49^. The herbivore fauna of the MPWP was potentially even richer than that of MIS2 and potentially exerted its impact far into the forested areas. This is suggested by, for example, fossil records of camel ancestors on Ellesmere Island (Canadian High Arctic) formerly covered by boreal larch forests^50^. The varying herbivore abundance, as inferred from the coprophilous *Sporormiella* spore record during the MPWP, may have led to marked vegetation variation. Our results suggest that herbivory during MPWP glacials turned a deciduous shrub-tundra (less *Betula nana* type (dwarf birch) in the pollen spectra, Supplementary Fig. 6f) into an open steppe forest (more *Larix* and Poaceae (grasses)) comparable to the ‘mammoth steppe' proposed for the LGM in Beringia^51^. A parallel decline of large herbivores and deciduous-shrub encroachment also occurs at the end of the late-glacial in North America^52^,^53^ and this pattern is supported by modern exclosure experiments^53^. Accordingly, our results indicate that herbivory may represent a significant vegetation driver on glacial–interglacial timescales acting independently of climate, thereby weakening the vegetation–climate link. However, no vegetation–herbivore relationship is indicated for the PPT, which contradicts the exploitation–ecosystem hypothesis^54^,^55^. According to this hypothesis unproductive ecosystems can only support food-limited grazers that exert a strong control on vegetation. More productive ecosystems such as occurred during the MPWP, in contrast, should be characterized by community-level trophic cascades, which should, according to this hypothesis, result in a weaker vegetation–herbivore relationship. However, the weak vegetation–herbivore ties during the PPT can potentially be explained by a local functional extinction of the megafauna, which was previously assumed when *Sporormiella* declined to <2% of the pollen sum^53^. However, it remains unknown if our observation of generally lower herbivore densities during the PPT compared with the MPWP represents a regional peculiarity or an Arctic-wide picture. Interestingly, the two major *Sporormiella* declines, that is, at the M2/M1 and G18/G17 transitions, occur when the magnitude of late-glacial warming was particularly strong. By analogy with the climate-driven extinction of some megaherbivores during the MIS2/Holocene transition^56^ we can only speculate that random processes such as extinctions during severe warming, at least partly, explain the observed herbivore density pattern around Lake El'gygytgyn. Furthermore, the potential refugia for cold-restricted plants and animals in the Asian Arctic are, compared with the North American Arctic, particularly small because the arctic coast is located further to the south and because the Bering Land Bridge was submerged during interglacials^40^, which may have enhanced random effects in interglacial colonization processes.

Our results suggest that vegetation–climate disequilibrium may have lasted for millennia. With the background of our results and the severity of the LGM (MIS2) we hypothesize that today's widely distributed larch forests lacking *Picea* and *Pinus* may not be in equilibrium with contemporary climate. Support for this comes from the fact that compared with *Larix*, which colonized Yakutia during the late-glacial, evergreen taxa expanded only during the early- and mid-Holocene^57^. However, they did not significantly extend their ranges beyond the present-day limit during the Holocene thermal maximum, in contrast to *Larix*^46^. Furthermore, evergreen taxa in southern and western Siberia probably represent late-successional vegetation in present-day larch-forest regions but they only become established when fire frequency is low^48^.

Vegetation records are commonly used to infer past temperature quantitatively, which is then compared with modelled or reconstructed atmospheric CO_2_ concentrations to assess Earth-system sensitivity^58^. When either the modern analogues or fossil records originate from vegetation in disequilibrium with climate, the inferred climate may be misleading. As expected from our results, the PPT temperature reconstruction based on Eĺgygytgyn pollen data^30^ poorly reflects the global SST^23^ and global ice-volume^22^ trends. This suggests that pollen-based quantitative climate reconstructions are less reliable than previously assumed, particularly for interglacials. However, independent regional climate records are necessary to quantify the uncertainties of pollen-based climate reconstructions. Likewise, only transient Earth-system simulations that include realistic long-term tree-population and permafrost dynamics and run over several glacial–interglacial cycles can resolve the timing and effectiveness of vegetation-related feedback mechanisms and thus provide realistic model-based estimates for Earth-system sensitivity.

With the appearance of permafrost and the functional disappearance of herbivory, the PPT at northern high latitudes represents a valuable natural experiment in a palaeoenvironmental setting with the potential to understand better the dynamics of Earth's past states when new system components are included.

## Methods

### Numerical analyses of original Lake El'gygytgyn pollen data

This paper uses a pollen data set from Lake El'gygytgyn^21^. Of the 1,166 samples that were analysed, 1,043 contained pollen. Of these, 844 samples with at least 150 terrestrial pollen grains and spores were included in the numerical analyses here. The pollen sum is >300 in most samples.

Principal curves^59^ were derived to best summarize, in a mathematical sense, the overall pollen compositional turnover and thus to maximize the variation represented in one dimension. To increase the signal-to-noise ratio, we *a priori* decided to include only half of the original 92 reliably recorded taxa (that is, taxa recorded at least twice in the entire data set); thus, all taxa with occurrences in >74 samples were retained for the principal curve analyses. Sample scores on the first correspondence axis were used as a starting curve. The principal curve was fitted using cubic smoothing splines allowing the complexity of the individual smoothers used in the local averaging step to vary between pollen taxa, using generalized cross-validation to select the optimal degree of smoothing for each taxon. A penalty term of 1.4 was used to increase the cost of degrees of freedom in the generalized cross-validation calculations^60^. Analyses were implemented in R using the pcurve package.

### Analyses of averaged interglacial and glacial pollen data

To obtain averaged interglacial and glacial data sets, principal curve minima and maxima were first assigned to marine isotope stages^22^ based on the original age model^20^. MIS84 was split into two glacial stages (MIS84a/c) and an interglacial stage (MIS84b) because a full glacial–interglacial turnover is clearly reflected in the pollen data. Boundaries between glacial and interglacials were set at the point where the mean principal curve value was crossed, calculated from the minimum principal curve values for interglacials and the maximum principal curve values for glacials. Original counts of those samples that are below/above the median PC values for each glacial/interglacial were then summed for all taxa. This step was done to ensure that the averaged data sets represent the ‘characteristic' pollen assemblages of each stage rather than the ‘transitional' assemblages between stages. To reduce the noise in the data set, percentages were calculated including vascular-plant pollen taxa that occur in at least 12 stages with a value of at least 2% once. Finally, a data set of 32 averaged interglacial pollen spectra and another of 32 averaged glacial pollen spectra were obtained.

Principal curve analysis of the combined glacial–interglacial averaged data set was run with a similar configuration as for the original data set, only the penalty term was set to 2 to account for the lower number of samples. Local-Contributions-To-Beta-Diversity^32^, that is, representing an estimation of the compositional uniqueness of a stage was likewise calculated using the combined averaged glacial–interglacial data set.

### Correlation analyses of global climate data sets

We analysed the global LR04 benthic inverted δ^18^O_stack_ (ref. 22) as well as a global collection of high-resolution SST records. We required that the full PPT (2,150–2,900 kyr ago) is covered with a mean resolution of at least 5 kyr per sample. Ten published UK^33^ records and the LR04 benthic stack meet these criteria (Supplementary Table 1).

The irregular raw data of every core were low-pass filtered using a Gaussian Kernel Smoother with an effective cutoff frequency of 1/15 kyr. The assignment of the glacial minima and interglacial maxima was performed in two steps. (1) Local maxima and minima were automatically identified from the smoothed time series. (2) All records were visually compared using their published age model, and the corresponding glacial minima and interglacial maxima were manually chosen from the minima and maxima of step 1. We only interpreted time periods where a 100-kyr window did not contain a data gap >15 kyr to reduce the possibility that we missed major peaks or troughs. In some cases, where no clear assignment was possible, no assignment was made; thus most records only contain a subset of all extremes. For ODP1082 and ODP1012, only a small number of maxima and minima could be assigned, probably due to the poor data quality of the records in the time period of interest. We thus excluded both these cores from further analysis. As the ODP982 SST record does not have a high enough resolution until 2,600 kyr ago, we correlated ODP982 and LR04 on an extended time window (2,600–3,500 kyr ago) to obtain a meaningful overlap with the LR04 stack. The results are summarized in Supplementary Fig. 4.

We also investigated whether the coherency between glacial and interglacial intensities was restricted to the marine SST records. For the last 800 kyr, we analysed several proxies derived from Antarctic ice cores^34^: water isotopes related to local temperature; a methane record potentially related to low-latitude wetland dynamics; and the CO_2_ record representing the global carbon cycle. We compared these records with the global benthic δ^18^O_stack_ (ref. 22) using the same method as in the previous section (Gaussian smoothing, automatic local extremes identification and manual assignment according to the marine isotope stages^22^) (Supplementary Fig. 5). We used the same technique of comparing local extremes instead of analysing the same points in time to be consistent with our main analyses of the Plio-Pleistocene time series.

### Redundancy analyses

Constrained ordination analyses were run to relate statistically the variation in the averaged glacial and interglacial pollen data sets to the proxy environmental variables. RDA was chosen over canonical correspondence analysis because of the compositional gradient lengths of the pollen data being <2 s.d. as revealed by initial detrended correspondence analyses, indicating that linear-based ordination methods are appropriate with these data sets. Because the boundary conditions strongly changed, we *a priori* separated the data of the MPWP (3,510–2,900 kyr ago, 12 glacials, 12 interglacials) from the PPT (2,900–2,150 kyr ago, 20 glacials, 20 interglacials) following definitions of the PLIOMAX group^61^. This boundary also accounts for the finding that the major decrease in atmospheric CO_2_ concentrations occurred around 2,900 kyr ago (ref. 62), that the phase of interglacials having higher than present sea-level ended^40^ and that ice-rafted debris markedly increased in glacial stages from MIS16 onwards^63^. Setting the boundary to that time also agrees with the results of the biome reconstruction using Lake El'gygytgyn pollen data, which indicates that MIS17 was the last interglacial that was at least temporarily dominated by cool coniferous forests^29^.

The climate proxy set includes the δ^18^O_stack_ record^22^ and two alkenone-based SST records from the Pacific as no other suitable regional climate proxy data are available. Total organic carbon (TOC) values or Si/Ti ratios from Lake El'gygytgyn were not used as proxy environmental variables because terrestrial vegetation changes could have strongly impacted lake chemistry^64^, thereby risking circular reasoning in our analyses. We selected the LR04 δ^18^O_stack_ record^22^, which, in addition to the temperature-sensitive ice-sheet extent, reflects the sea-level position and thus variability in continentality in northern Asia. Furthermore, the proxy set includes the high-resolution alkenone-based SST record from the northern mid-latitude ODP site 1208 in the Pacific^30^. This SST record represents the closest record to Lake El'gygytgyn and is located near to the potential glacial refugia in north-eastern Asia and has, furthermore, a high data quality. Because the SST record from ODP site 1208 does not fully cover the MPWP the alkenone-based SST from the eastern Pacific ODP846 site^23^, which is well correlated to ODP site 1208 during the PPT^65^, is used for analysing the MPWP. The δ^18^O stack and SST records were interpolated to 2.5 kyr ago time slices and the maximum/minimum value for each interglacial/glacial was used. The same proxy records were used as variables to represent the preceding stage climate.

Square-root-transformed values of *Sporormiella* spore percentages (relative to the vascular-plant pollen sum), a coprophilous fungus commonly occurring in the palynomorph record, were used as a herbivore intensity proxy^31^. Likewise, percentages of *Gelasinospora* spores, which are often found at times of massive charcoal input^66^,^67^, were used as a fire-frequency proxy. Support for this comes from the observation that the occurrence of *Gelasinospora* is significantly related to burnt soils^26^, which is explained by its rapid growth rate and the resistance of its spores to high temperatures compared to other fungi. Preceding stage PC values of the combined glacial/interglacial averaged data set were used to explore long-term vegetation drivers. Furthermore, percentages of *Glomus* spores were used to indicate changes in the disturbance and erosion of soils^28^.

The following sets of RDAs were run on each of the Hellinger-transformed glacial and interglacial averaged pollen data sets. Those variables (or variable sets), which when solely included in an initial RDA yielded a statistically significant relationship with the data set (*P*<0.1), were included together in variation partitioning to extract the uniquely explained variation by each single environmental variable (or variable set) and the shared explained variation. Explained variations represent adjusted *r*^2^ values^68^.

### Analyses of PPT interglacial vegetation histories

We aimed to investigate whether pollen inter-taxa relationships among PPT interglacials with preceding warm glacials (that is, ‘warm interglacials') are different from those among interglacials with preceding cold glacials (that is, ‘cold interglacials'). For that purpose, warm interglacials (that is, having a combination of low δ^18^O_stack_ and high SST values compared with all interglacials during the PPT, and having a minimum of 12 samples: G9, G7, G3, MIS101 and MIS93) and cold glacials (that is, having a combination of high δ^18^O_stack_ and low SST, and having a minimum of 12 samples: G1, MIS103, MIS99, MIS95 and MIS83) were selected based on the climate proxy information and the minimum sample number. Inter-taxa relationships of a single interglacial are reflected by the species scores in an ordination. Accordingly, principal component analyses (with scaling focused on variable correlations) were performed on the original pollen samples of the respective warm or cold interglacials. Analyses were based on Hellinger-transformed pollen percentages of taxa that occur at least three times in each stage. To compare the ordination results of two interglacials the correlation of variable scores on the first four principal component analysis (PCA) axes was assessed using Procrustes analysis^69^ and its associated Protest test^70^. Finally, the Protest *P* values gained from pair-wise comparisons among all ‘warm interglacials' and among all ‘cold interglacials' were presented in boxplots.

### Statistical programmes used and data availability

All analyses were implemented in R version 3.03 using functions in packages analogue 0.12-0, calibrate 1.7.2, pcurve 0.6-5, vegan 2.0-10 and the function beta.div. Averaged data sets and environmental proxy data used for redundancy analyses are available at www.pangaea.de.

## Additional information

**How to cite this article:** Herzschuh, U. *et al*. Glacial legacies on interglacial vegetation at the Pliocene-Pleistocene transition in NE asia. *Nat. Commun.* 7:11967 doi: 10.1038/ncomms11967 (2016).

## Supplementary Material

Supplementary Information Supplementary Figures 1-10, Supplementary Tables 1-3, Supplementary Note 1 and Supplementary References.

## References

[b1] Shugart H. H. & Woodward F. I. Global Change and the Terrestrial Biosphere Wiley-Blackwell (2011).

[b2] Vazquez-Rivera H. & Currie D. J. Contemporaneous climate directly controls broad-scale patterns of woody plant diversity: a test by a natural experiment over 14 000 years. Global Ecol. Biogeogr. 24, 97–106 (2014).

[b3] Blois J. L., Williams J. W., Fritzpatrick M. C., Jackson S. T. & Ferrier S. Space can substitute for time in predicting climate-change effects on biodiversity. Proc. Natl. Acad. Sci. USA 110, 9374–9379 (2013). 23690569 10.1073/pnas.1220228110 PMC3677423

[b4] Svenning J.-C. Deterministic Plio-Pleistocene extinctions in the European cool-temperate tree flora. Ecol. Lett. 6, 646–653 (2003).

[b5] Smith A. G. Problems of inertia and threshold related to post-glacial habitat changes. Proc. R. Soc. London B 161, 331–342 (1965).

[b6] Prentice I. C., Bartlein P. J. & Webb T. Vegetation and climate change in eastern North-America since the Last Glacial Maximum. Ecology 72, 2038–2056 (1991).

[b7] Giesecke T. . The pace of Holocene vegetation change—testing for synchronous developments. Quat. Sci. Rev. 30, 2805–2814 (2011).

[b8] Svenning J.-C. & Sandel B. Disequilibrium vegetation dynamics under future climate change. Am. J. Bot. 100, 1266–1286 (2014). 23757445 10.3732/ajb.1200469

[b9] Tzedakis P. C. & Bennett K. D. Interglacial vegetation succession: a view from southern Europe. Quat. Sci. Rev. 14, 967–982 (1995).

[b10] Cheddadi R. . Similarity of vegetation dynamics during interglacial periods. Proc. Natl. Acad. Sci. USA 102, 13939–13943 (2005). 16162676 10.1073/pnas.0501752102 PMC1236528

[b11] Normand S. . Postglacial migration supplements climate in determining plant species range in Europe. Proc. R. Soc. B 278, 3644–3653 (2011). 10.1098/rspb.2010.2769 PMC3203492 21543356

[b12] Blonder B. . Linking environmental filtering and disequilibrium to biogeography with a community climate framework. Ecology 96, 972–985 (2015). 26230018 10.1890/14-0589.1

[b13] Miller G. H. . Arctic amplification: can the past constrain the future? Quat. Sci. Rev. 29, 1779–1790 (2010).

[b14] Vandenberghe J. . The Last Permafrost Maximum (LPM) map of the Northern Hemisphere: permafrost extent and mean annual air temperatures, 25-17 ka BP. Boreas 43, 652–666 (2014).

[b15] Owen-Smith R. N. Megaherbivores: The Influence of Very Large Body Size on Ecology Cambridge Univ. Press (1988).

[b16] Gill J. L. Ecological impacts of the late Quaternary megaherbivore extinctions. New Phytol. 201, 1163–1169 (2014). 24649488 10.1111/nph.12576

[b17] Bond W. J. & Keeley J. E. Fire as a global ‘herbivore': the ecology and evolution of flammable ecosystems. Trends Ecol. Evol. 20, 387–394 (2005). 16701401 10.1016/j.tree.2005.04.025

[b18] Komárková V. Vegetation type hierarchies and landform disturbance in arctic Alaska and alpine Colorado with emphasis on snowpatches. Vegetatio 106, 155–181 (1993).

[b19] Kienast F. . Paleontological records indicate the occurrence of open woodlands in a dry inland climate at the present-day Arctic coast in western Beringia during the Last Interglacial. Quat. Sci. Rev. 30, 2134–2159 (2011).

[b20] Brigham-Grette J. . Pliocene warmth, polar amplification and stepped Pleistocene cooling recorded in NE Arctic Russia. Science 340, 1421–1427 (2013). 23661643 10.1126/science.1233137

[b21] Andreev A. A. . Late Pliocene and Early Pleistocene environments of the north-eastern Russian Arctic inferred from the Lake El'gygytgyn pollen record. Clim. Past 10, 1017–1039 (2014).

[b22] Lisiecki L. E. & Raymo M. E. A Pliocene-Pleistocene stack of 57 globally distributed benthic δ^18^O records. Paleoceanography 20, PA1003 (2005).

[b23] Lawrence K. T., Liu Z. & Herbert T. D. Evolution of the Eastern Tropical Pacific through Plio-Pleistocene Glaciation. Science 312, 79–83 (2006). 16601186 10.1126/science.1120395

[b24] Prescott C. L. . Assessing orbitally-forced interglacial climate variability during the mid-Pliocene Warm Period. Earth Planet. Sci. Lett. 400, 261–271 (2014).

[b25] Commandini O. & Rinaldi A. C. Tracing megafaunal extinctions with dung fungal spores. Mycologist 18, 140–142 (2004).

[b26] Widden P. & Parkinson D. The effects of a forest fire on soil microfungi. Soil Biol. Biochem. 7, 125–138 (1975).

[b27] Anderson R. S., Homola R. L., Davis R. B. & Jacobson G. L. Fossil remains of the mycorrhizal fungal *Glomus fasciculatum* complex in postglacial lake sediments from Maine. Can. J. Bot. 62, 2325–2328 (1984).

[b28] Van Geel B. in Tracking Environmental Change Using Lake Sediments, vol. 3: Terrestrial, Algal and Silicaceous Indicators (eds Smol, J. P. Birks H. J. B., Last W. M. 99–119Kluwer (2001).

[b29] Tarasov P. E. . A pollen-based biome reconstruction over the last 3.562 million years in the Far East Russian Arcti—new insights into climate–vegetation relationships at the regional scale. Clim. Past 9, 2759–2775 (2013).

[b30] Venti N. L., Billups K. & Herbert T. D. Increased sensitivity of the Plio-Pleistocene northwest Pacific to obliquity forcing. Earth Planet. Sci. Lett. 384, 121–131 (2013).

[b31] Isaev A. P. . in The Far North: Plant Biodiversity and Ecology of Yakutia (eds Troeva, E. I. .) 143–260Springer (2010).

[b32] Legendre P. & DeCaceres M. Beta diversity as the variance of community data: dissimilarity coefficients and partitioning. Ecol. Lett. 16, 951–963 (2013). 23809147 10.1111/ele.12141

[b33] Lloyd A. Ecological histories from Alaskan tree lines provide insight into future change. Ecology 86, 1687–1695 (2005).

[b34] Masson-Delmotte V. . EPICA Dome C record of glacial and interglacial intensities. Quat. Sci. Rev. 29, 113–128 (2010).

[b35] Mitchell J. M. . An overview of climatic variability and its causal mechanisms. Quat. Res. 6, 481–493 (1976).

[b36] Jones P. D., Osborn T. J. & Briffa K. R. Estimating sampling errors in large-scale temperature averages. J. Clim. 10, 2548–2568 (1997).

[b37] Willeit M., Ganopolski A., Calov R., Robinson A. & Maslin M. The role of CO_2_ decline for the onset of Northern Hemisphere glaciation. Quat. Sci. Rev. 119, 22–34 (2015).

[b38] Osawa A., Zyryanova O. A., Matsuura Y., Kajimoto T., Wein R. W. (eds) Permafrost Ecosystems: Siberian Larch Forests Springer 502 (2010).

[b39] French H. The Periglacial Environment Wiley478 p (2007).

[b40] Miller K. G., Mountain G. S., Wright J. D. & Browning J. V. A 180-millon-year record of sea level and ice volume variations from continental margin and deep-sea isotopic records. Oceanography 24, 40–53 (2011).

[b41] Polyak L. . History of sea ice in the Arctic. Quat. Sci. Rev. 29, 1757–1778 (2010).

[b42] Reyes A. V., Froese D. G. & Jensen B. J. L. Permafrost response to last interglacial warming: field evidence from non-glaciated Yukon and Alaska. Quat. Sci. Rev. 29, 3256–3274 (2010).

[b43] Jiang Y., Zhuang Q. & O'Donnell J. A. Modeling thermal dynamics of active layer soils and near-surface permafrost using a fully coupled water and heat transport model. J. Geophys. Res. 117, D11110 (2012).

[b44] Galushkin Y. Numerical simulation of permafrost evolution as a part of sedimentary basin modelling: permafrost in the Pliocene-Holocene climate history of the Urengoy field in the West Siberian basin. Can. J. Earth Sci. 34, 935–948 (1997).

[b45] Demske D., Mohr B. & Oberhänsli H. Late Pliocene vegetation and climate of Lake Baikal region, southern East Siberia, reconstructed from palynological data. Palaeogeogr. Palaeoclim. Palaeoecol. 184, 107–129 (2002).

[b46] Polezhaeva M., Laxcoux M. & Semerikov V. L. Cytoplasmic DNA variation and biogeography of *Larix* Mill. in Northeast Asia. Mol. Ecol. 19, 1239–1252 (2010). 20163546 10.1111/j.1365-294X.2010.04552.x

[b47] Harden J. W., Manies K. L., Neff J. C. & Turetsky M. R. Effects of wildfire and permafrost on soil organic matter and soil climate in interior Alaska. Global Change Biol. 12, 1–13 (2006).

[b48] Schulze E.-D. . Factors promoting larch dominance in central Siberia: fire versus growth performance and implications for carbon dynamics at the boundary of evergreen and deciduous conifers. Biogeosciences 9, 1405–1421 (2012).

[b49] Gill J. L., Williams J. W., Jackson S. T., Donnelly J. P. & Schellinger G. C. Climatic and megaherbivory controls on late-glacial vegetation dynamics: a new high-resolution, multi-proxy record from Silver Lake, Ohio. Quat. Sci. Rev. 34, 66–80 (2012).

[b50] Rybczynski N. . Mid-Pliocene warm-period deposits in the High Arctic yield insight into camel evolution. Nat. Commun. 4, 1550 (2013). 23462993 10.1038/ncomms2516 PMC3615376

[b51] Blinnikov M. S., Gaglioti B., Walker D. A., Wooller M. J. & Zazula G. D. Pleistocene graminoid-dominated ecosystems in the Arctic. Quat. Sci. Rev. 30, 2906–2929 (2011).

[b52] Gill J. L., Williams J. W., Jackson S. T., Lininger K. B. & Robinson G. S. Pleistocene megafaunal collapse, novel plant communities, and enhanced fire regimes in North America. Science 326, 1100–1103 (2009). 19965426 10.1126/science.1179504

[b53] Bakker E. S. . Combining paleo-data and modern exclosure experiments to assess the impact of megafauna extinctions on woody vegetation. Proc. Natl. Acad. Sci. USA 113, 847–855 (2016). 26504223 10.1073/pnas.1502545112 PMC4743795

[b54] Oksanen L. & Oksanen T. The logic and realism of the hypothesis of exploitation ecosystems. Am. Nat. 155, 703–723 (2000). 10805639 10.1086/303354

[b55] Anapuu M. . Spatial pattern and dynamic responses of Arctic food webs corroborate the exploitation ecosystems hypothesis (EEH). Am. Nat. 171, 249–262 (2008). 18197777 10.1086/524951

[b56] Lorenzen E. . Species-specific responses of Late Quaternary megafauna to climate and humans. Nature 479, 359–364 (2011). 22048313 10.1038/nature10574 PMC4070744

[b57] Binney H. A. . The distribution of late-Quaternary woody taxa in northern Eurasia: evidence from a new macrofossil database. Quat. Sci. Rev. 28, 2445–2464 (2009).

[b58] Schmittner A. . Climate sensitivity estimated from temperature reconstructions of the Last Glacial Maximum. Science 334, 1385–1388 (2011). 22116027 10.1126/science.1203513

[b59] De'ath G. Principal curves: a new technique for indirect and direct gradient analysis. Ecology 80, 2237–2253 (1999).

[b60] Simpson G. L. & Birks H. J. B. in Tracking Environmental Change Using Lake Sediments, vol. 5: Data Handling and Numerical Techniques (eds Birks, H. J. B., Lotter, A. F., Juggins, S. & Smol, J. P.) 249–327Springer (2012).

[b61] Pliomax Group http://pliomax.org/pliowiki/index.php/Welcome_to_PLIOMAX.

[b62] Seki O., Foster G. L., Schmidt D. N. & Mackensen A. Alkenone and boron-based Pliocene pCO_2_ records. Earth Planet. Sci. Lett. 292, 201–211 (2010).

[b63] Flesche Kleiven H., Jansen E., Fronval T. & Smith T. M. Intensification of Northern Hemisphere glaciations in the circum-Atlantic region (3.5–2.4 Ma)—ice-rafted detritus evidence. Palaeogeogr. Palaeoclim. Palaeoecol. 184, 213–223 (2002).

[b64] Herzschuh U. . Siberian larch forests and the ion content of thaw-lakes form a geochemically functional entity. Nat. Commun. 4, 2408 (2013). 24005763 10.1038/ncomms3408

[b65] Nie J. . Surface-water freshening: a cause for the onset of the North Pacific stratification from 2.75 Ma onward? Global Planet. Change 64, 49–52 (2008).

[b66] Anderson R. S., Ejarque A., Brown P. M. & Hallett D. J. Holocene and historical vegetation change and fire history on the north-central coast of California, USA. Holocene 23, 1797–1810 (2013).

[b67] Froyd C. A. . The ecological consequences of megafaunal loss: giant tortoises and wetland biodiversity. Ecol. Lett. 17, 144–154 (2014). 24382356 10.1111/ele.12203 PMC4015371

[b68] Peres-Neto P., Legendre P., Dray S. & Borcard D. Variation partitioning of species data matrices: estimation and comparison of fractions. Ecology 87, 2614–2625 (2006). 17089669 10.1890/0012-9658(2006)87[2614:vposdm]2.0.co;2

[b69] Peres-Neto P. & Jackson D. A. How well do multivariate sets match? The advantages of a Procrustean superimposition approach over the Mantel test. Oecologia 129, 169–178 (2001). 10.1007/s004420100720 28547594

[b70] Jackson D. A. PROTEST: a Procrustes randomisation test of community environment concordance. Ecoscience 2, 297–303 (1995).

