# Supplementary material for: Glacial legacies on interglacial vegetation at the Pliocene-Pleistocene transition in NE Asia
Source: Nat Commun. 2016 Jun 24;7:11967. doi: 10.1038/ncomms11967 (PMC4931021; doi:10.1038/ncomms11967)
Supplement: Supplementary Information — Supplementary Figures 1-10, Supplementary Tables 1-3, Supplementary Note 1 and Supplementary References. [file ncomms11967-s1.pdf]

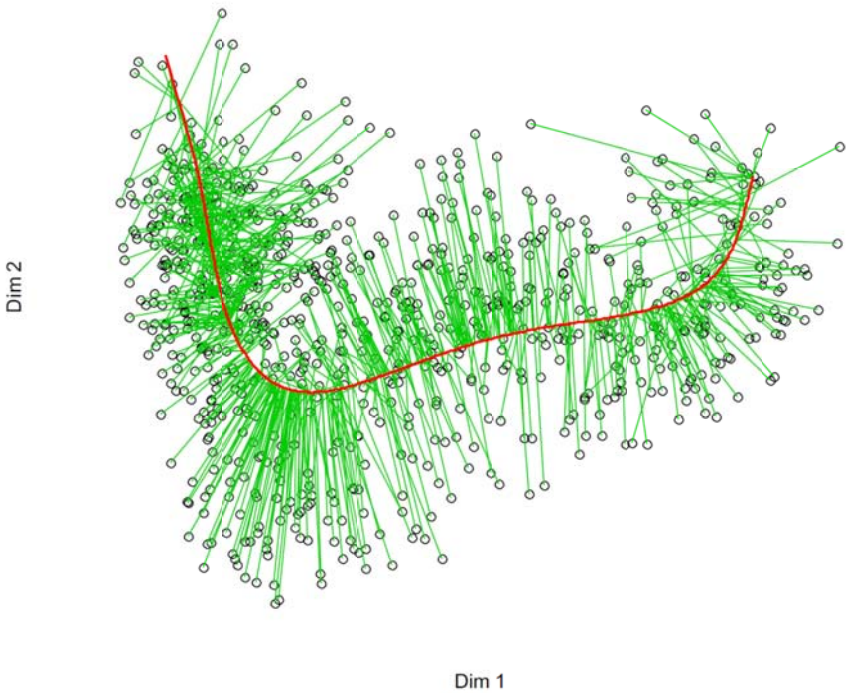


**Supplementary Fig. 1 Results from prinicipal curve analyses.** Fitted principal curve (PC: red line) of the Lake El´gygytgyn original pollen data projected onto the first two dimensions in PCA space. The green lines relate each observation with its position on the PC. PCA (Dim) axis 1 is the horizontal axis and PCA (Dim) axis 2 is the vertical axis. In total, 47% of variation in the pollen data are explained by the PC.

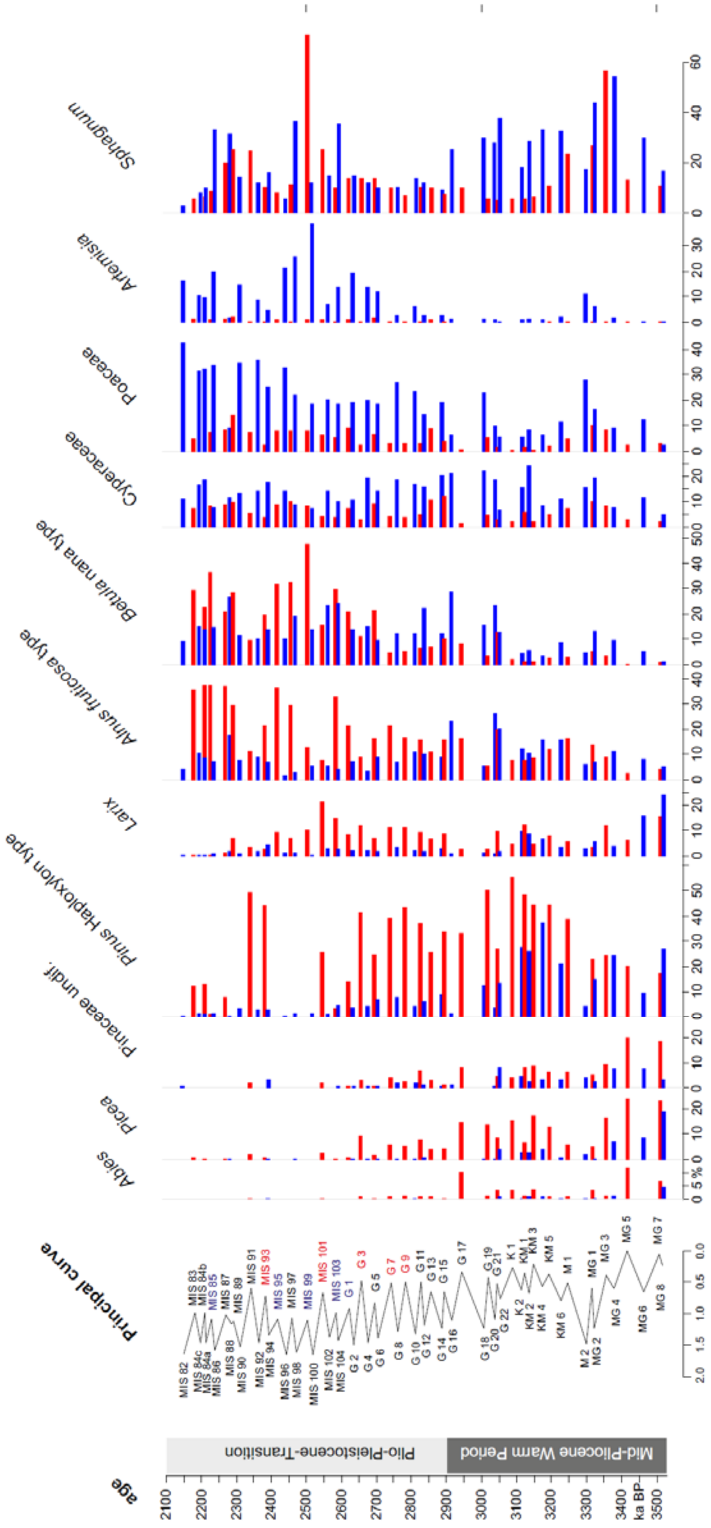


**Supplementary Fig. 2 Pollen diagram.** Only those taxa are shown that are included in the principal curve analysis (PCA) of averaged glacial and interglacial stage data-sets. Percentages are calculated based on the pollen sum of these taxa. *Sphagnum* is plotted for comparison. Red indicates interglacial spectra; blue refers to glacial spectra.

a)

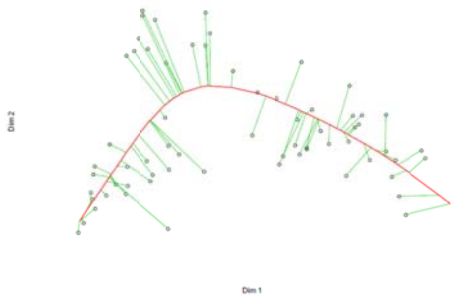


b)

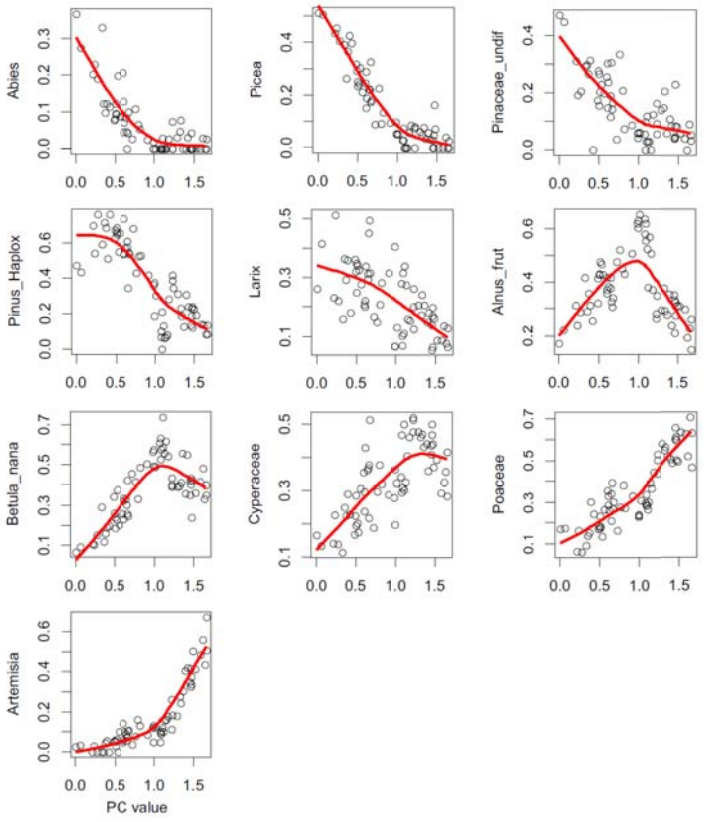


**Supplementary Fig. 3 Results of principal curve analysis of glacial/interglacial data set.** a) Fitted principal curve (PC: red line, explaining 75% of variation in the pollen data) a) of the Lake El´gygytgyn averaged stage pollen data-set projected onto the first two dimensions (Dim) in PCA space. The green lines relate each observation with its position on the PC. b) Fitted response curves of each pollen taxon. Open circles are the observed proportional abundance along their principal curve locations and the solid red line is the optimised smoother from the final iteration of the principal curve. The taxa are arranged according to visual inspection of their response curves optima (from right to left and from top to bottom). The vertical axis is the Hellinger-transformed pollen taxon abundance and the horizontal axis is distance along the principal curve.

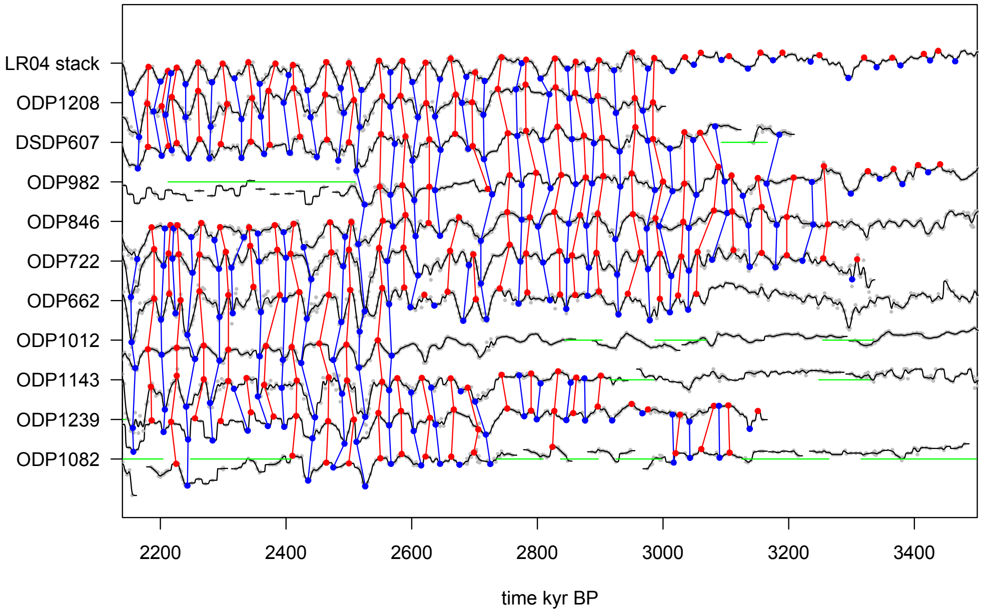


**Supplementary Fig. 4 Overview of all Uk37records and the identified minima and maxima.** Grey dots show the raw data-points, black lines the smoothed time-series, and blue and red dots are the identified glacial minima and interglacial maxima, respectively. Green horizontal lines show the time-periods where the resolution is too low for analysis. The LR04 $\delta^{18}O_{stack}$ represents inverted values. Note that the interglacials in ODP722, ODP1143, ODP662, and ODP1239 are close to the warm-temperature limit of the Uk37 proxy (warmer than 27°C) leading to a larger uncertainty in the interglacial temperature estimates[1].

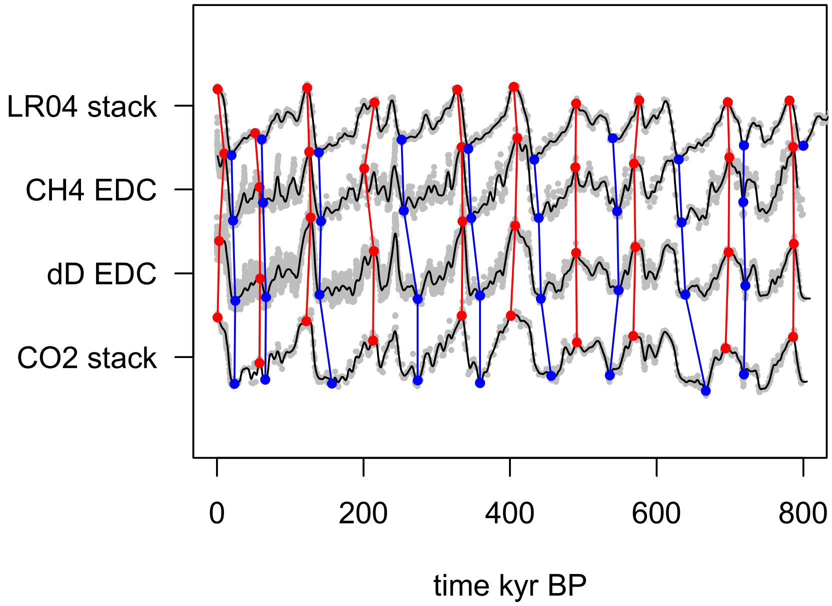


**Supplementary Fig. 5 Overview of all records and the identified minima and maxima for the analysis of the last 800 kyr.** Grey dots show the raw data-points, black lines the smoothed time-series, and blue and red dots are the identified glacial minima and interglacial maxima, respectively. Data-sets used are EDC δD[2], EDC $CH_4$ [3], $CO_2$ composite record[4], all on the AICC12 chronology[5] and the benthic $\delta^{18}O_{stack}$[6]. The LR04 $\delta^{18}O_{stack}$ represents inverted values.

Interglacials of the Pliocene Pleistocene Transiton

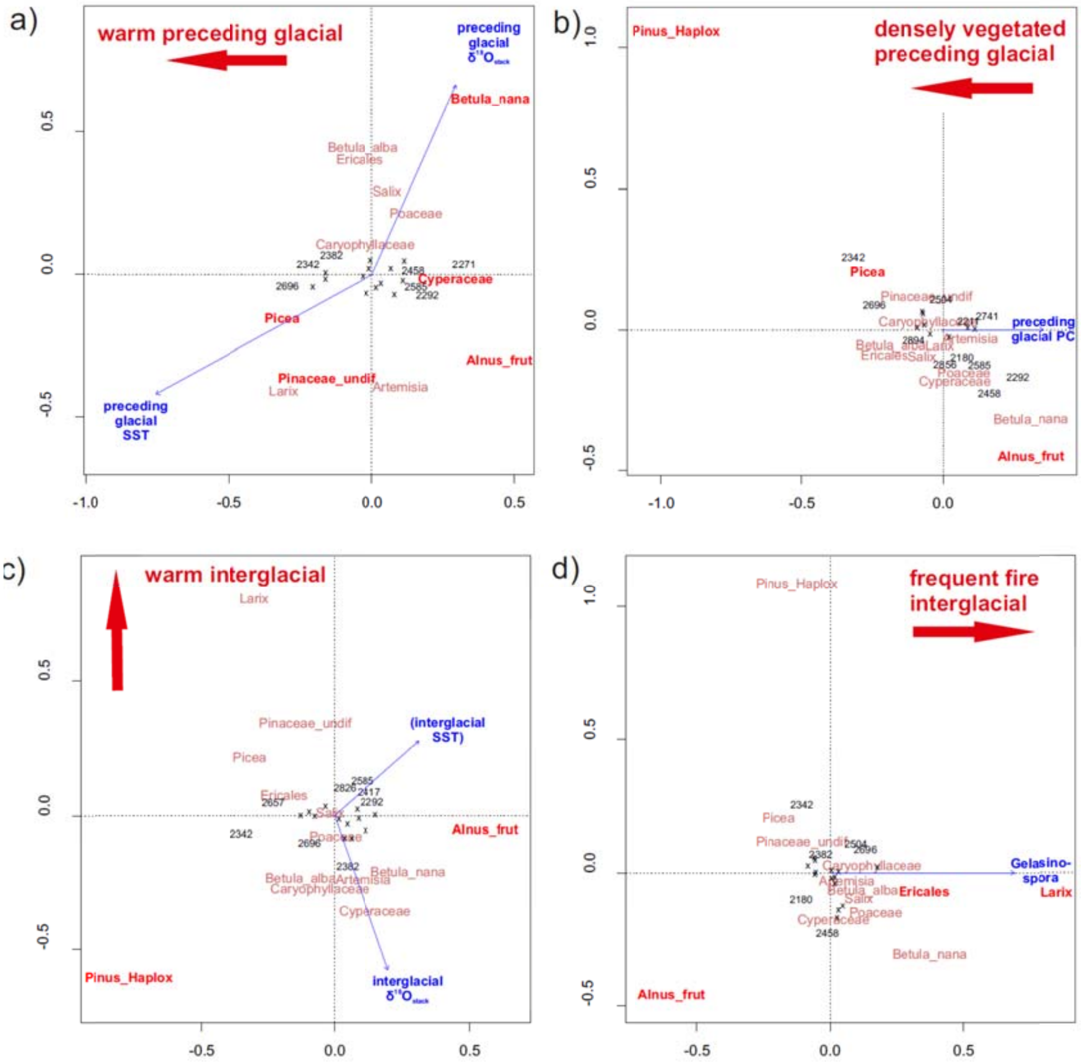


**Supplementary Fig. 6 Results of variation partitioning using RDA.** Plots of the first two axes (RDA axis 1 is horizontal, RDA axis 2 is vertical) from variation partitioning using redundancy analyses (RDA). For sub-plots a-d the averaged interglacial pollen data-set of the Plio-Pleistocene Transition (PPT) is used. For sub-plots e-f the averaged glacial pollen data-set of the Mid-Pliocene Warm Period (MPWP) is used. The plots refer to the RDA results using the following settings: a) unique variation in the PPT interglacial pollen data-set explained by preceding stage climate after extracting the variation explained by climate, fire, and preceding stage vegetation); b) unique variation in the PPT interglacial pollen data-set explained by preceding stage vegetation after extracting the variation explained by climate, fire, and preceding stage climate; c) unique variation in the PPT interglacial pollen data-set explained by climate after extracting the variation explained by fire, preceding stage climate, and preceding stage vegetation; d) unique variation in the PPT interglacial pollen data-set explained by fire after extracting the variation explained by climate, preceding stage climate, and preceding stage vegetation. Explained variation in the pollen data is indicated in Fig. 4 of the main text. Red arrows and related text highlight the environmental conditions indicated by the proxy data-sets used as constraints in RDAs. Non-significant environmental variables are in brackets. Taxa marked in bold are significantly ($p$<0.1) correlated with the first constrained axis. Only selected interglacials are indicated for purposes of clarity.

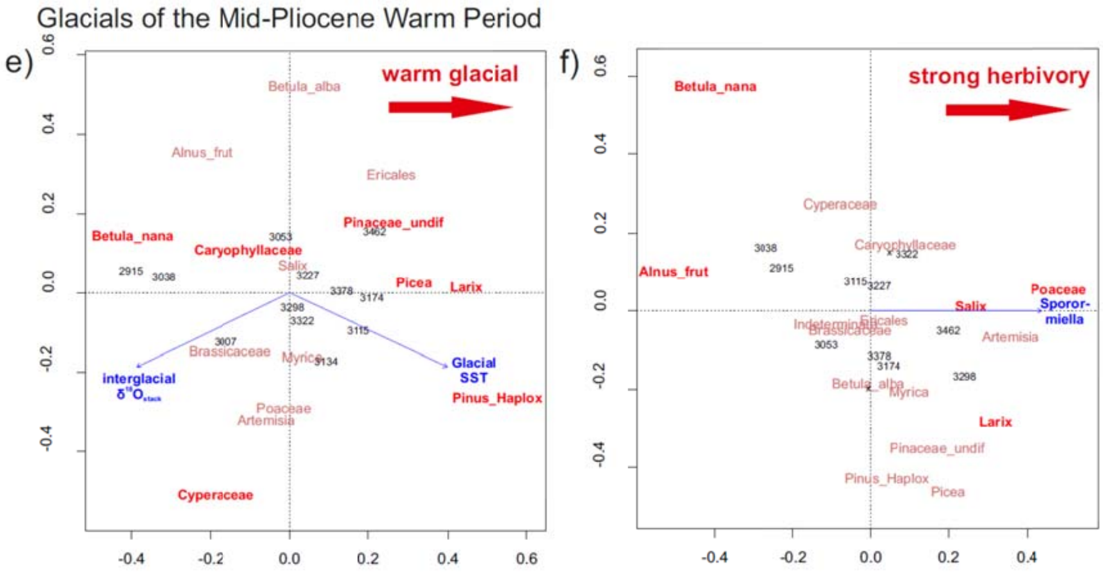


**Continuation of Supplementary Fig. 6**

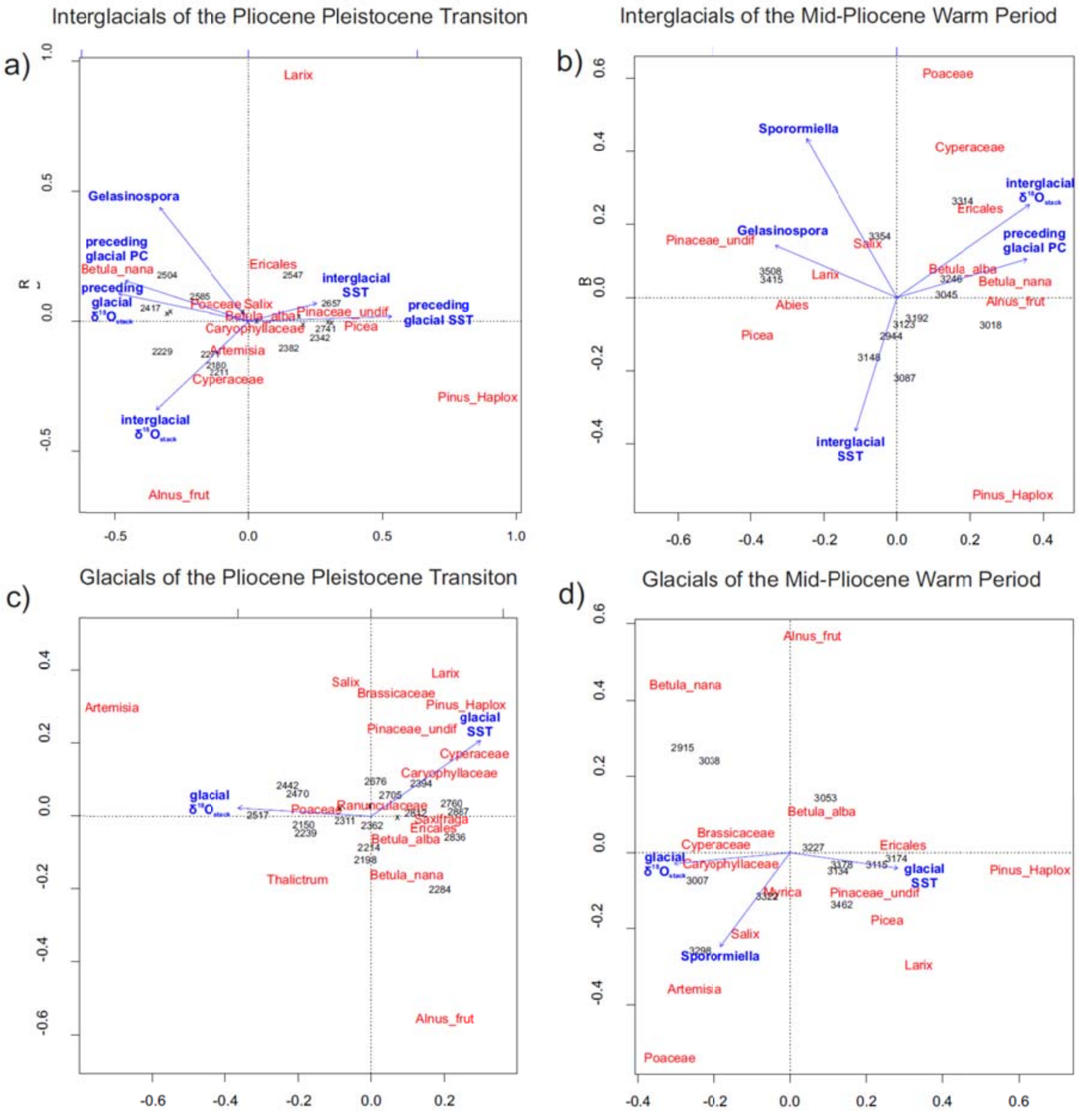


**Supplementary Fig. 7 Results of redundancy analyses.** Plots of the first two axes of redundancy analyses (RDA axis 1 is horizontal, RDA axis 2 is vertical) performed involving the four average stage data-sets (a – Plio-Pleistocene transition (PPT) interglacials, b – Mid-Pliocene Warm Period (MPWP) interglacials, c – PPT interglacials, d – MPWP glacials) and jointly including those environmental variables (i.e. those for climate, fire, herbivory, previous stage climate, and previous stage vegetation) that explain a significant portion of variation in the pollen data when included in separate RDAs (see Supplementary Table 2 lower part).

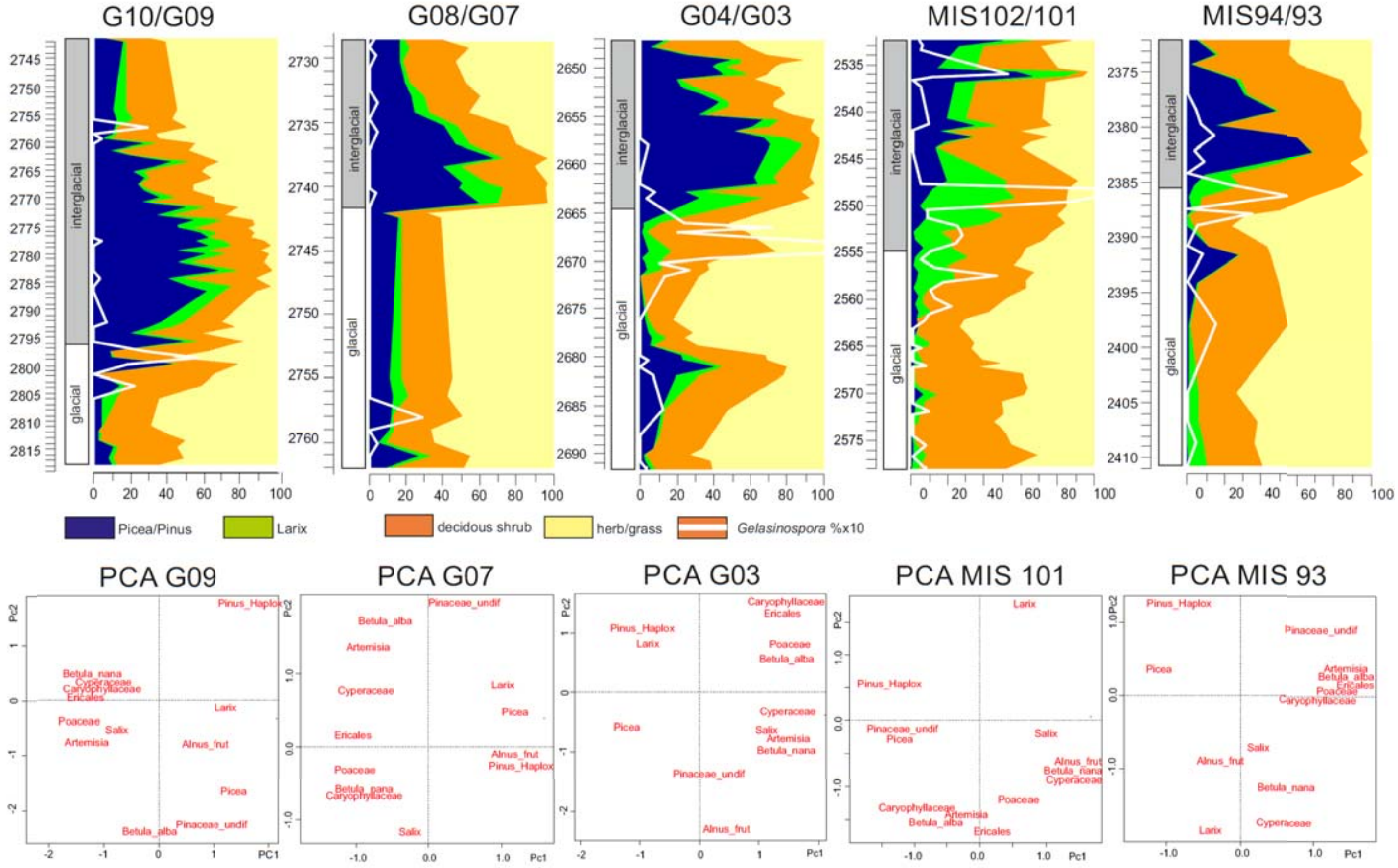


**Supplementary Fig. 8 Summary information of glacial/interglacial cycles with warm glacial stages.** Summary pollen diagrams of glacial/interglacial cycles with a warm glacial stage used for inter-comparison of interglacial inter-taxa relationships and, below each pollen diagram, the related taxa plot for the first two principal component analysis (PCA) axes.

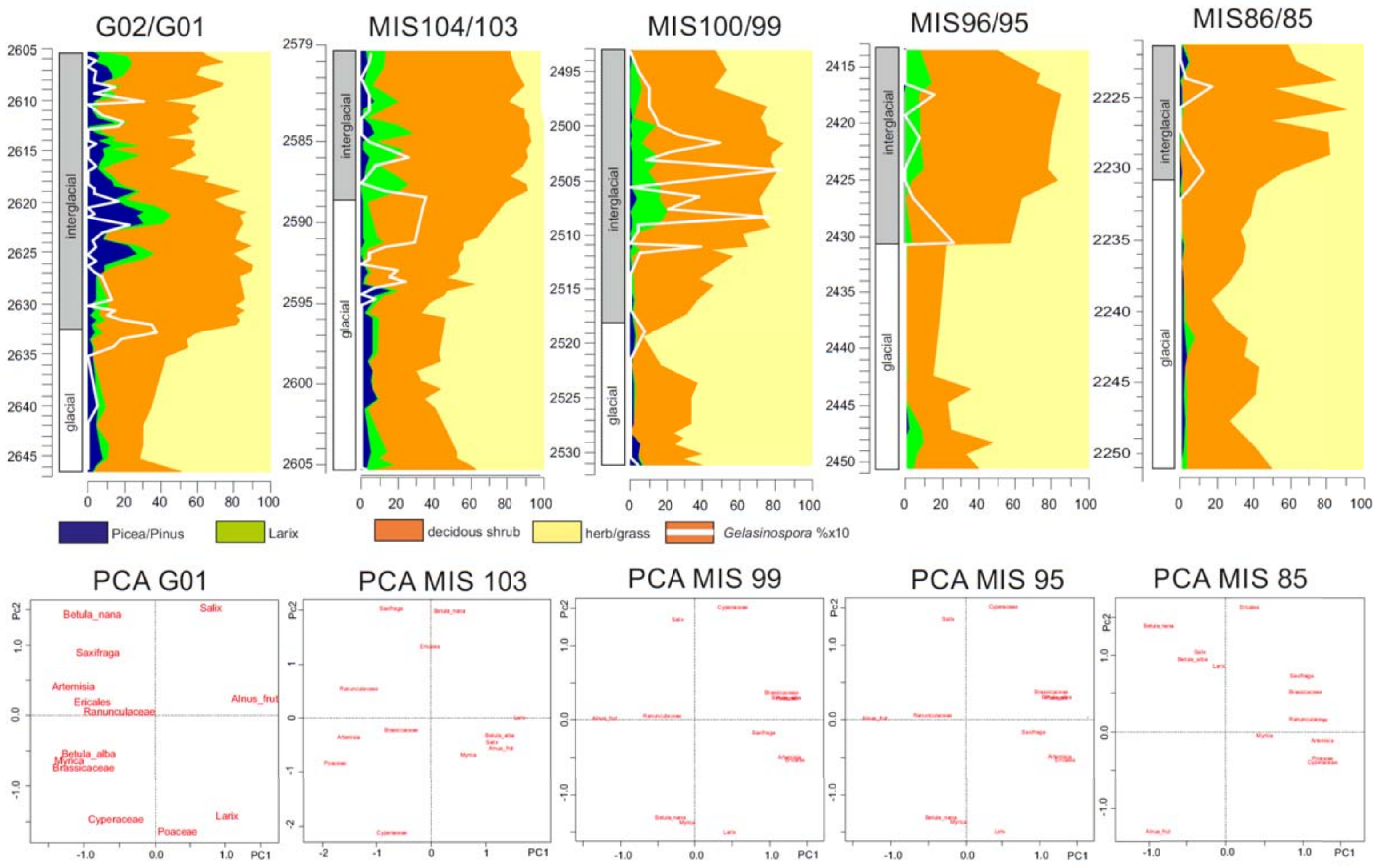


**Supplementary Fig. 9 Summary information of glacial/interglacial cycles with cold glacial stages.** Summary pollen diagrams of glacial/interglacial cycles with a cold glacial stage used for inter-comparison of interglacial inter-taxa relationships and, below each pollen diagram, the related taxa plot for the first two principal component analysis (PCA) axes.

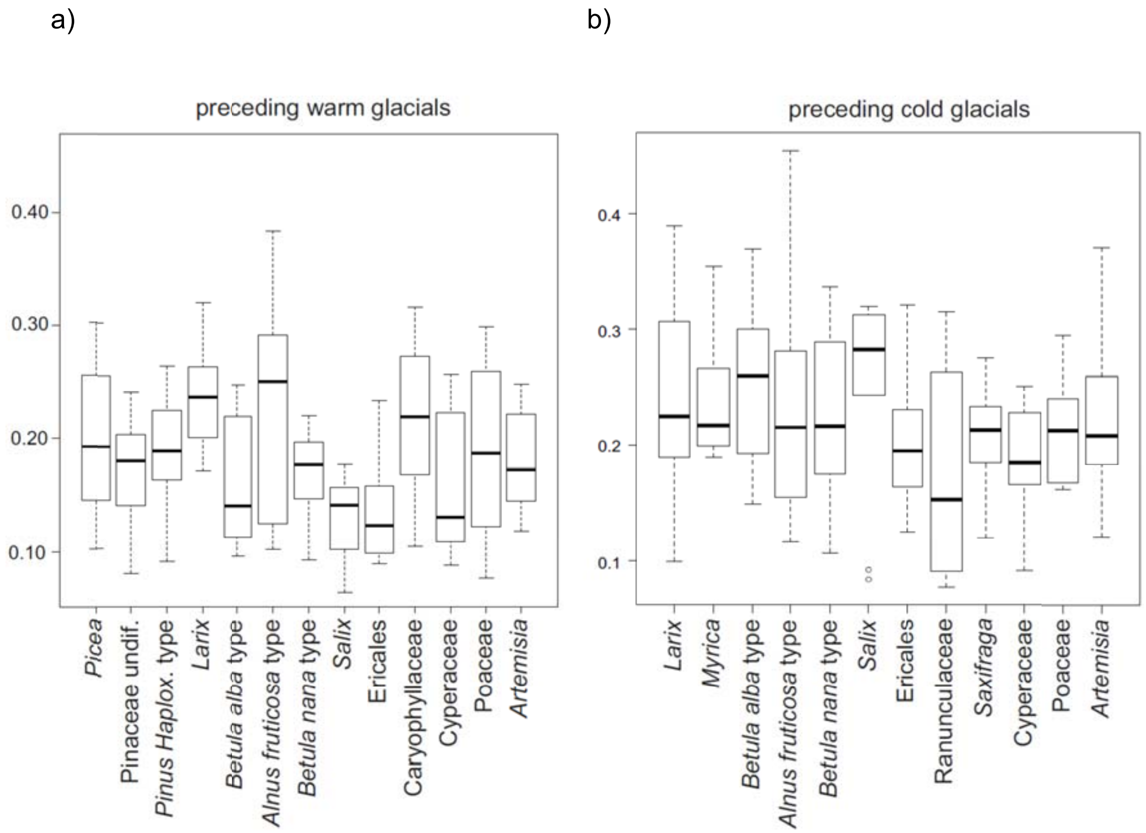


**Supplementary Fig. 10 Results of Procrustes analyses.** Boxplots of the Procrustes residuals for each taxon from pairwise comparisons among Plio-Pleistocene transition (PPT) interglacials with a preceding warm glacial stage (a)) that in almost all pairwise comparisons produce a significant fit (as revealed by the results from the Protest analyses, see Fig. 5 of main text); and with a preceding cold glacial stage (b)) that in almost all cases produce a non-significant fit. Whiskers represent the 1.5-time length of interquartile range. Results indicate that *Larix* and deciduous shrubs have the highest residuals. This indicates that these taxa obtain high abundances in assemblages with unique compositions.

**Supplementary Table 1 Core-sites used for the global intercomparison of glacial and interglacial intensities.** We only retain Uk37 records as Mg/Ca records potentially suffer variations due to seawater Mg/Ca on the time-scales of interest[7]. Mean resolution and sea-surface temperature (SST) range were evaluated in our core-period of interest 2900–2100 ka. Red marks temperature ranges above 27°C. (Note that for one position, in the North Atlantic, two records exist, DSDP6078[8] and U1313[9]. We decided to analyse only DSDP607 as both records have a similar resolution at the Plio-Pleistocene transition, but the alkenone record of DSDP607 is measured with the more reliable GC-FID technique, and the age model of DSDP607 is based on benthic oxygen isotopes, a parameter not available for U1313.)

| **Core Name** | **Lat** (°N) | **Lon** (°E) | **Mean resolution** (kyr) | **$SST_{Uk37}$ range** (°C) |
|---|---|---|---|---|
| ODP1082[10] | -21.1 | 11.8 | 3.9 | 19.6-26.7 |
| ODP1239[11] | -0.7 | -82.1 | 4.8 | 24.0-27.9 |
| ODP1143[12] | 9.4 | 113.3 | 2.5 | 27.2-29.0 |
| ODP1012[13] | 32.3 | -118.4 | 2.2 | 13.5-23.0 |
| ODP662[14] | -1.4 | -11.7 | 2.0 | 23.5-27.4 |
| ODP722[14] | 16.6 | 59.8 | 2.0 | 24.4-28.0 |
| ODP846[15] | -3.1 | -90.8 | 2.1 | 21.2-26.4 |
| ODP982[16] | 57.5 | -15.9 | 4.8 | 10.5-18.3 |
| DSDP607[8] | 41.0 | -33.0 | 3.0 | 13.8-21.8 |
| ODP1208[17] | 36.1 | 158.2 | 2.3 | 14.9-22.4 |
| $\delta^{18}O_{stack}$[6] | global | Global | 2.5 | NA |

**Supplementary Table 2**

**Results from redundancy analyses.** Percentage of explained variation, i.e. from adjusted $r^2$ in averaged pollen data-sets of the Pliocene and Pleistocene glacials and interglacials using selected environmental variables or variable sets. The lower part indicates the percentage of explained variation when each climate variable is included as a single variable in RDA. (Asterisks represent *p*-values of adjusted $r^2$: ***<0.001, **<0.01, *<0.05, .<0.1).

| | **Explained variance (%)** | | | |
|---|---|---|---|---|
| | Pliocene Interglacials (N=12) | Pliocene Glacials (N=12) | Pleistocene-like Interglacials (N=20) | Pleistocene-like Glacials (N=20) |
| **Variables or variable sets used** | | | | |
| Climate ($\delta^{18}O_{stack}$ + SST) | 21.59* | 18.32* | 14.85. | 25.50** |
| Herbivory (sqrt *Sporormiella*%) | 15.04* | 12.03* | 0.82 | 1.51 |
| Fire (sqrt *Gelasinospora*%) | 12.67* | 4.27 | 17.54* | 3.59 |
| Disturbance (sqrt *Glomus*%) | 6.47 | 0 | 0 | 7.90* |
| Preceding stage climate ($\delta^{18}O_{stack}$+ SST) | 3.92 | 0.51 | 41.97*** | 5.17 |
| Preceding stage vegetation (preceding stage PC value) | 15.22* | 6.07 | 29.38** | 3.00 |
| **Single variables** | | | | |
| SST | 4.32 | 16.08** | 6.25 | 17.07*** |
| $\delta^{18}O_{stack}$ | 19.40** | 19.63** | 17.28* | 25.91*** |
| SST of preceding stage | 0.67 | 0.77 | 39.99*** | 2.49 |
| $\delta^{18}O_{stack}$ of preceding stage | 9.44 | 4.86 | 32.28** | 0.14 |

sqrt = square root, PC = principal curves, SST = sea-surface temperature.

**Supplementary Table 3 Results from redundancy analyses using temperature estimates from various SST records.** Percentage of uniquely and jointly explained variation (i.e. from adjusted $r^2$ in averaged pollen data-sets of Plio-Pleistocene transition (PPT) interglacials using climate variables for the contemporaneous interglacial climate and preceding glacial climate). (Asterisks represent *p*-values of adjusted $r^2$: ***<0.001, **<0.01, *<0.05, .<0.1).

| Core name | Number of inter-glacials | Contemporary interglacial climate | Preceding glacial climate | Together |
|---|---|---|---|---|
| $\delta^{18}O_{stack}$ | 20 | 5.7 | 22.4** | 38.6*** |
| SST ODP1208 | 20 | 0 | 27.6** | 35.6*** |
| SST ODP1239 | 18 | 0 | 29.1*** | 50.8*** |
| SST ODP1143 | 18 | 0 | 27.2** | 28.9** |
| SST ODP662 | 20 | 0 | 19.9** | 23.1* |
| SST ODP722 | 19 | 0 | 14.2* | 16.3* |
| SST ODP846 | 18 | 6.3 | 21.5** | 39.9** |
| SST DSDP607 | 20 | 4.6 | 25.9** | 46.0*** |

SST = sea-surface temperature

## Supplementary Note 1

**Discussion about the occurrence of *Pinus pumila*.** *Pinus pumila* is able to grow on shallow permafrost soils and is a common element in the Russian Far East mountains, particularly in the maritime and snow-rich Russian Far East[18]. However, we assume that the major part of the *Pinus* Haploxylon pollen-type is related to tree pine taxa and not to the shrub *Pinus pumila* for the following reasons. First, *Pinus* Haploxylon-type is highly correlated with *Picea* pollen (PPT interglacials: $r$=0.80, $p$-value = 2.708e-05, entire data-set: $r$=0.58, $p$-value = 4.014e-07) and not with other permafrost-tolerant shrubs such as *Alnus fruticosa*-type (PPT interglacials: $r$=0.09, $p$-value = 0.6413; entire data-set: $r$=0.029, $p$-value = 0.8187). Second, *Pinus pumila* is observed to be a low pollen producer[19] in contrast to tree pine which is known to have very high pollen productivity. Third, today *Pinus pumila* has a preference for an oceanic climate with abundant snow. Because at least during the Plio-Pleistocene transition, sea-level was not markedly higher but in most periods markedly lower than today[20] it is unlikely that *Pinus pumila* could attain a higher cover during that period than today when it is rather rare in the Lake El´gygytgyn area.

## Supplementary references


1 Grimalt, J.O., Calvo, E. & Pelejero, C. Sea surface paleotemperature errors in Uk′37 estimation due to alkenone measurements near the limit of detection. *Paleoceanography* **16**, 226–232 (2001).

2 Jouzel, J. et al. Orbital and millennial Antarctic climate variability over the past 800,000 years. *Science* **317**, 793–796 (2007).

3 Loulergue, L. et al. Orbital and millennial-scale features of atmospheric $CH_4$ over the past 800,000 years. *Nature* **453**, 383–386 (2008).

4 Lüthi, D. et al. High-resolution carbon dioxide concentration record 650,000–800,000 years before present. *Nature* **453**, 379–382 (2008).

5 Veres, D. et al. The Antarctic ice core chronology (AICC2012): an optimized multi-parameter and multi-site dating approach for the last 120 thousand years. *Clim. Past* **8**, 1733-1748 (2013).

6 Lisiecki, L.E. & Raymo, M.E. A Pliocene-Pleistocene stack of 57 globally distributed benthic δ 18 O records: *Paleoceanography* **20**, PA1003. doi: 10.1029/2004PA001071 (2005).

7 Medina-Elizalde, M., Lea, D. W. & Fantle, M. S. Implications of seawater Mg/Ca variability for Plio-Pleistocene tropical climate reconstruction. *Earth Planet. Sci. Lett*. **269**, 585–595 (2008).

8 Lawrence, K.T., Sosdian, S., White, H.E. & Rosenthal, Y. North Atlantic climate evolution through the Plio-Pleistocene climate transitions. *Earth Planet. Sci. Lett*. **300**, 329–342 (2010).

9 Naafs, B.D. A. et al. Strengthening of North American dust sources during the late Pliocene (2.7 Ma). *Earth Planet. Sci. Lett*. **317–318**, 8–19 (2012).

10 Etourneau, J., Martinez, P., Blanz, T. & Schneider, R. Pliocene–Pleistocene variability of upwelling activity, productivity, and nutrient cycling in the Benguela region. *Geology* **37**, 871–874 (2009).

11 Etourneau, J., Schneider, R., Blanz, T. & Martinez, P. Intensification of the Walker and Hadley atmospheric circulations during the Pliocene–Pleistocene climate transition. *Earth Planet. Sci. Lett*. **297**, 103–110 (2010).

12 Li, L. et al. A 4-Ma record of thermal evolution in the tropical western Pacific and its implications on climate change. *Earth Planet. Sci. Lett*. **309**, 10–20 (2011).

13 Brierley, C.M. et al. Greatly expanded tropical warm pool and weakened Hadley circulation in the early Pliocene. *Science* **323**, 1714–1718 (2009).

14 Herbert, T.D., Peterson, L.C., Lawrence, K.T. & Liu, Z. Tropical ocean temperatures over the past 3.5 million years. *Science* **328**, 1530–1534 (2010).

15 Lawrence, K.T., Liu, Z. & Herbert, T.D. Evolution of the eastern tropical Pacific through Plio-Pleistocene glaciation. *Science* **312**, 79–83 (2006).

16 Lawrence, K.T., Herbert, T.D., Brown, C.M., Raymo, M.E. & Haywood, A.M. High-amplitude variations in North Atlantic sea surface temperature during the early Pliocene warm period. *Paleoceanography* **24**, PA2218 (2009).

17 Venti, N.L., Billups, K. & Herbert, T.D. Increased sensitivity of the Plio-Pleistocene northwest Pacific to obliquity forcing. *Earth Planet. Sci. Lett*. **384**, 121–131 (2013).

18 Isaev, A.P., et al., Vegetation of Yakutia: Elements of ecology and plant sociology. In Troeva, I.E. et al. (eds). *The Far North: Plant Biodiversity and Ecology of Yakutia*. pp. 143-260. Springer (2010).

19 Binney, H.A., et al., The distribution of late-Quaternary woody taxa in northern Eurasia: evidence from a new macrofossil database. *Quat. Sci. Rev.* **28**, 2445-2464 (2009).

20 Miller, K.G., Mountain, G.S., Wright, J.D., Browning, J.V.. A 180-millon-year record of sea level and ice volume variations from continental margin and deep-sea isotopic records. *Oceanography* **24**, 40-53 (2011).
